# Unraveling the role of ZNF506 as a human PBS-pro-targeting protein for ERVP repression

**DOI:** 10.1093/nar/gkad731

**Published:** 2023-09-11

**Authors:** Qian Wu, Lu Fang, Yixuan Wang, Peng Yang

**Affiliations:** Shanghai Key Laboratory of Anesthesiology and Brain Functional Modulation, Clinical Research Center for Anesthesiology and Perioperative Medicine, Translational Research Institute of Brain and Brain-Like Intelligence, Shanghai Fourth People's Hospital, School of Life Sciences and Technology, Tongji University, Shanghai 200092, China; Shanghai Key Laboratory of Anesthesiology and Brain Functional Modulation, Clinical Research Center for Anesthesiology and Perioperative Medicine, Translational Research Institute of Brain and Brain-Like Intelligence, Shanghai Fourth People's Hospital, School of Life Sciences and Technology, Tongji University, Shanghai 200092, China; Translational Medical Center for Stem Cell Therapy & Institute for Regenerative Medicine, Shanghai East Hospital, School of Life Sciences and Technology, Tongji University, Shanghai 200092, China; Shanghai Key Laboratory of Anesthesiology and Brain Functional Modulation, Clinical Research Center for Anesthesiology and Perioperative Medicine, Translational Research Institute of Brain and Brain-Like Intelligence, Shanghai Fourth People's Hospital, School of Life Sciences and Technology, Tongji University, Shanghai 200092, China

## Abstract

Krüppel-associated box zinc finger proteins (KZFPs) function as a defense mechanism to maintain the genome stability of higher vertebrates by regulating the transcriptional activities of transposable elements (TEs). While previous studies have characterized ZFP809 as responsible for binding and repressing ERVs containing a proline tRNA primer-binding site (PBS-Pro) in mice, comparable KZFPs have not been identified in humans yet. Here, we identified ZNF506 as a PBS-Pro-binding protein in humans, which functions as a transcriptional repressor of PBS-Pro-utilizing retroviruses by recruiting heterochromatic modifications. Although they have similar functions, the low protein similarities between ZNF506, ZFP809 and KZFPs of other species suggest their independent evolution against the invasion of PBS-Pro-utilizing retroviruses into their respective ancestor genomes after species divergence. We also explored the link between ZNF506 and leukemia. Our findings suggest that ZNF506 is a unique human KZFP that can bind to PBS-Pro, highlighting the diverse evolution of KZFPs in defending against retroviral invasions. Additionally, our study provides insights into the potential role of ZNF506 in leukemia, contributing to the expanding knowledge of KZFPs' crucial function in disease and genome stability.

## Graphical Abstract

**Graphical Abstract:**
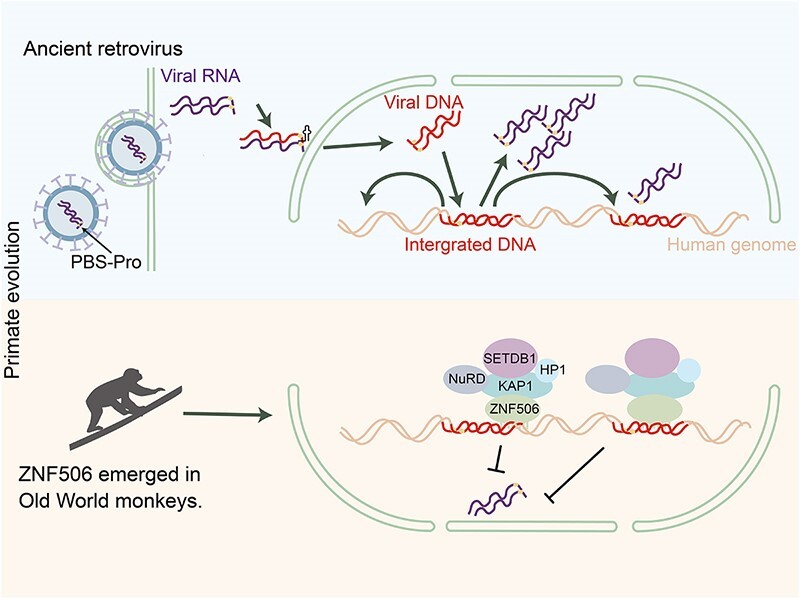

## INTRODUCTION

During evolution, organisms have generated various defense mechanisms to resist the threats of exogenous and endogenous retroviruses (ERVs), protecting genome stability and host safety. As important members of the defense system in higher vertebrates, Krüppel-associated box (KRAB) zinc finger (ZF) proteins (KZFPs) have rapidly evolved and expanded to be the largest family of transcriptional regulators in mice and humans (1), regulating the expression of transposable elements (TEs) in the genome at the transcriptional level (2). Although they are diverse, members of KZFPs exhibit similar characteristics in their structure. The KRAB domains at the N-terminus have been shown to recruit the scaffold structural protein KAP1 (also known as TRIM28) (3,4), which promoted heterochromatic modifications and transcriptional repression by further recruitment of repressive factors, including the histone methyltransferase SETDB1 (also known as ESET) (5), the nucleosome remodeling and deacetylation (NRD/HDAC) complex (6), heterochromatin protein 1 (HP1) (7) and DNA methyltransferases (8). The tandem repeats of C2H2-ZFs at the C-terminus are shown to specifically recognize and bind to the targeted DNA sequences (9,10). Typically, the four amino acids at positions −1, 2, 3 and 6 in the C2H2 α-helix of each ZF, referred to as the key zinc ‘fingerprint’ amino acids, are responsible for direct interaction with three consecutive nucleotides of the target DNA strand, thus determining the specificity of ZF binding (1,11–13), and have been adopted as important parameters for computational prediction of KZFP-targeting sequences (14).

Large-scale chromatin immunoprecipitation (ChIP)-seq experiments on human cells revealed the preferred binding of KZFPs to TEs (15,16). An important insight into the evolution and function of KZFPs in higher vertebrates was the finding that the number of KZFP genes in different species was correlated with long terminal repeat (LTR) transposons (17). Increasing evidence shows that KZFPs and TEs appeared simultaneously in higher vertebrates (18), and the rapidly mutating TEs evolved to escape the transcriptional repression of KZFPs, which in turn impelled the continuous evolution of the KZFP gene pool (2,19). The evolutionary arms race between KZFPs and TEs amplified the diversity of host genes, complicated the interacting networks and drove selection. In addition, the dynamic competition between KZFPs and TEs benefited the host through spatiotemporal regulation (20,21), which further promoted the development and evolution of mammals (22).

ERVs are remnants of ancient exogenous retroviral invasions into the genome (23). They retain many conservative elements of exogenous viruses, including the primer-binding site (PBS) (24). These PBS elements, located at the LTR region and composed of 18 nucleotides, serve as the recognition sites for host tRNAs and facilitate the initiation of retroviral reverse transcription (25). While diverse retrovirus families display unique types of PBS, the sequences within a specific retroviral family are generally conserved (24). To date, several types of PBS have been identified, including PBS-Lys, PBS-Leu and PBS-Pro (24,25).

In mice, ZFP809 was the first KZFP discovered to specifically bind to PBS sequences of ERVs (10,26–28). ChIP-seq analysis revealed that ZFP809 can specifically recognize PBS-Pro sequences, thereby recruiting KAP1 and SETDB1 to silence VL30-pro and integrated DNA of the exogenous retrovirus MLV through H3K9me3 modifications (10). Recent studies have identified ZFP961 as a PBS-Lys-binding protein, which represses the ERVK subfamily by targeting the PBS-Lys sequences (29).

The genome-wide KZFP ChIP-seq data generated from 293T cells have also provided a valuable resource for studying KZFP in humans (16), enabling the identification of human KZFPs that target different types of PBS sequences. Among these, ZNF417 and ZNF587 have been demonstrated to repress human endogenous retrovirus K (HERVK) and human immunodeficiency virus 1 (HIV-1) by targeting the PBS-Lys sequences (30,31).

Considering the substantial presence of ERVs with PBS-Pro sequences in the human genome (23,24), as well as the utilization of PBS-Pro sequences by exogenous retroviruses such as human T-lymphotropic virus (HTLV) during replication (32), identifying KZFPs capable of binding to PBS-Pro sequences in humans is of great significance, which could potentially serve as a restriction factor against retroviruses. As the PBS-Pro-binding protein ZFP809 is mouse specific and lacks orthologs in the human genome (28), it is speculated that primate-specific KZFPs with similar functions may have evolved independently to confer resistance against ERVP invasion.

In this study, we have identified and confirmed ZNF506 as a repressor of the ERVP subfamily in humans by targeting the PBS-Pro sequences. We also assessed the repressive effects of ZNF506 on the infectivity of PBS-Pro-utilizing retroviruses introduced exogenously into cells. In addition, our evolutionary analysis demonstrated that the emergence of ZNF506 in the human genome coincided with the invasion of the ERVP subfamily, supporting the hypothesis of an evolutionary arms race between KZFPs and ERVs. Although both ZNF506 and ZFP809 target PBS-Pro, they exhibit distinct binding preferences and utilize different ZF domains. We propose that different species have developed unique countermeasures against retroviral invasions, contributing to the non-homologous evolution of KZFPs. Furthermore, we discuss the potential role of KZFPs, particularly ZNF506, in leukemia, where their aberrant expression may influence disease progression. Our study highlights the role of the species-specific gene ZNF506 in binding and repressing the ERVP subfamily, showcasing the potential significance of their interplay in the context of acute myeloid leukemia (AML).

## MATERIALS AND METHODS

### Calculation of appearance time of KZFPs

Authentic orthologs for each KZFP were obtained from the Ensembl database (33), which was used to calculate the age of each KZFP. The age of each KZFP was determined by the evolutionary divergence time from the species that have the oldest ortholog to humans. Data on species divergence times were obtained from TimeTree (34). The similarities between genes and the similarities between the regions near the genes were taken into account during the calculation. The reliability of the calculation was also marked to better reflect the distribution of gene orthologs across species.

For other species, we also used Uniprot annotations to identify the KZFPs present in these species. Due to the lack of ortholog data for these KZFPs, we used the annotations from UniRef90 to approximate the possible orthologs. Specifically, for each KZFP in *Ovis aries*, *Ailuropoda melanoleuca*, *Balaenoptera musculus*, *Myotis lucifugus* and *Panthera leo*, we adopted the annotation results on UniRef90 to determine those proteins with >90% similarities to the KZFPs, which served as possible orthologs of the KZFPs. Using these orthologs and the TimeTree data on the timing of species divergence, we estimated the emergence time of each KZFP in these five species.

### Timing for ERVs to invade species genomes

For humans, we used the data of ERVs in the human genome calculated by previous studies (24), including the sequence information of each component in the ERVs and the classification information of the ERV families. For the other five species, we downloaded the latest reference genomes and used the RetroTector (35) to search for ERVs *de novo* in these species, thus obtaining the information on ERVs in these species.

For the subfamily of ERVPs containing PBS-Pro, its insertion time was calculated by using the divergence of LTR elements (a divergence of 0.4% corresponds to 1 million years). By analyzing the distribution information of the insertion time of the ERVP subfamily, we can obtain the peak period of their replication in the species genome. KZFPs with inhibitory effects on this subfamily of ERVs should be produced during this period.

We first screened the KZFPs that co-occurred with the ERVP subfamily using the invasion time of the ERVP subfamily calculated above and the appearance time of KZFPs in each species. Among them, there may be KZFPs that have evolved to resist the invasion and replication of the ERVP subfamily by targeting the PBS-Pro sequences.

### Screening for KZFPs capable of binding to PBS-Pro sequences

To identify KZFPs capable of binding to PBS-Pro sequences in the human genome, we employed a multi-step screening process. Firstly, we utilized the RetroTector (35) software to extensively explore the intact ERVs within the human genome, obtaining their corresponding PBS types and confidence scores. To ensure the accuracy of the PBS classification by the software, we adopted the methodology from a previous study (24) to obtain the high-confidence PBS-Pro candidates (scores > 100), which led to the identification of 46 credible PBS-Pro annotations.

Out of a total of 348 distinct KZFPs identified within the human genome, ChIP-seq experimental data were available for 236 KZFPs (16,36). We then analyzed these 236 KZFPs, focusing on their binding peaks relative to the calculated PBS-Pro regions. Notably, ZNF506 displayed significant binding affinity towards PBS-Pro, while other KZFPs exhibited distinct binding motifs distinct from PBS-Pro.

To search for additional KZFPs with potential PBS-Pro binding activity among the remaining 112 KZFPs without reported ChIP-seq data, we first screened for KZFPs corresponding to the timing of ERVP invasions and identified 17 candidate KZFPs. Next, we analyzed the similarity between the DNA-binding motifs of these candidates, as predicted by the DNA binding prediction using the Zinc Finger Recognition Code (http://zifrc.ccbr.utoronto.ca/), and the PBS-Pro sequences. Despite these efforts, we failed to identify any promising candidates. Furthermore, we compared the fingerprint profiles of these 112 KZFPs with those of ZNF506 and ZFP809 via sequence alignment. We observed a low degree of similarity (< 50%). These results indicate that the interaction between ZNF506 and PBS-Pro sequences might be unique among human KZFPs.

### Cell culture and generation of cell lines

293T cells were cultured in Dulbecco’s modified Eagle’s medium (DMEM) high glucose (Gibco) containing 10% fetal bovine seum (FBS; Gibco) and 2 mM l-glutamine (Millipore). KZFP cDNAs were cloned in a piggyBac cytomegalovirus (CMV)–green fluorescent protein (GFP) shuttle vector that produced GFP-fused proteins. ZNF506–GFP and ZNF417–GFP vectors were stably transfected into 293T cells and co-transferred with the piggyBac transposon vector in a 1:1 ratio. A BD FACS Aria II was used to isolate single GFP-positive cells for subsequent expansion, and the cell lines were cultured in 1 μg/ml puromycin. pX330 plasmids encoding hCas9 were purchased from Addgene (#42230), and single guide RNAs (sgRNAs) were cloned into the BbsI restriction site. To construct the *ZNF506* knockout (KO) cell lines, pX330 and GFP vectors were co-transfected into 293T cells following the instructions for a Lonza 4D-Nucleofector. A BD FACS Aria II was used to isolate single GFP-positive cells for subsequent expansion and genotyping.

### Luciferase assays

For motif binding assays, annealed oligos (different PBS sequences) were cloned downstream of the pGL3-SV40 vector (Promega) using In-Fusion cloning (Tiangen). The pGL3 reporter vectors were co-transfected with different KZFP genes in piggyBac CMV–GFP vectors into 293T cells using VigoFect (Vigorous) together with the *Renilla* luciferase-expressing pRL-SV40p vector (Addgene #27163) for internal normalization. Luciferase activity was measured 48 h after transfection using the dual-luciferase reporter assay system (Promega).

### ChIP-seq and ChIP-qPCR analysis

ZNF506–GFP 293T cell lines and *ZNF506* KO cell lines were used to perform ChIP experiments with specific antibodies [GFP (Invitrogen A-6455) for KZFPs; and KAP1 (Abcam ab22553) and H3K9me3 (Abcam ab8898) for histone marks], and ZNF417–GFP 293T cell lines were used as controls for H3K9me3. For ChIP experiments, 2–4 × 10^7^ cells were cross-linked for 10 min at room temperature by the addition of a formaldehyde solution at a final concentration of 1% followed by quenching with glycine. Cells were washed twice with phosphate-buffered saline, then the supernatant was aspirated, and the cell pellets were stored at −80°C. Pellets were then lysed, resuspended in 1 ml of LB1 [50 mM HEPES-KOH pH 7.4, 140 mM NaCl, 1 mM ethylenediamine tetraacetic acid (EDTA), 0.5 mM glycol ether diamine tetraacetic acid (EGTA), 10% glycerol, 0.5% NP-40, 0.25% Triton X-100 and protease inhibitors] on ice for 10 min, and after centrifugation resuspended in LB2 (10 mM Tris pH 8.0, 200 mM NaCl, 1 mM EDTA, 0.5 mM EGTA and protease inhibitors) on ice for 10 min. After centrifugation, cells were resuspended in LB3 [10 mM Tris pH 8.0, 200 mM NaCl, 1 mM EDTA, 0.5 mM EGTA, 0.1% sodium deoxycholate, 0.1% sodium dodecylsulfate (SDS) and protease inhibitors] for histone marks and SDS shearing buffer (10 mM Tris pH 8, EDTA 1 mM, 0.15% SDS and protease inhibitors) for transcription factors, and then sonicated (Covaris settings: 5% duty, 200 cycle, 140 PIP, 20 min), yielding genomic DNA fragments with an average size of 100–300 bp. Beads were coated with specific antibodies during the day at 4°C, and then chromatin was added to the beads overnight at 4°C for histone marks, while antibodies for transcription factors were first incubated with chromatin in 1% Triton X-100 and 150 mM NaCl. Subsequently, washes were performed with 2× low salt wash buffer (10 mM Tris pH 8, 1 mM EDTA, 150 mM NaCl and 0.15% SDS), 1× high salt wash buffer (10 mM Tris pH 8, 1 mM EDTA, 500 mM NaCl, 0.15% SDS), 1× LiCl buffer (10 mM Tris pH 8, 1 mM EDTA, 0.5 mM EGTA, 250 mM LiCl, 1% NP-40 and 1% sodium deoxycholate) and once with TE buffer. DNA was purified using a QIAGEN elution column. The ChIPed DNA was then subjected to next-generation sequencing or quantitative polymerase chain reaction (qPCR) analysis. For ChIP-seq, up to 10 ng of ChIPed DNA or input DNA (Input) was subjected to sequencing library preparation. Sequencing was performed on a NovaSeq 6000 (Illumina) following library construction using a KAPA HyperPrep Kit (Roche). Next, 150 bp paired-end reads per ChIP-seq were mapped to the human (hg38) genome using Bowtie2 (37) with default parameters. Peaks were called using MACS analysis software using Input as the control and specifying *P*-values < 10^−6^ as significant (38). Consensus target motifs were derived from peak regions using MEME-ChIP (https://meme-suite.org/meme/tools/meme-chip). KZFP binding peak analysis was performed using BEDtools (39), and the matrix between ChIP peaks and repeats was calculated using ComputeMatrix. Heatmaps and profile plots were then generated by plotHeatmap or plotProfile using the calculated matrix generated by ComputeMatrix (40).

For ChIP-qPCR to test the enrichments of RNA polymerase II (Pol II), H3K9me3 and H3K27ac on pMX virus LTR regions, ZNF506–GFP and ZNF417–GFP 293T cell lines were infected with pMX-RFP pseudovirus, respectively. Cells were harvested and cross-linked after 48 h of infection and subjected to ChIP with anti-RNA Pol II (CTD4H8, Millipore), anti-H3K9me3 (Abcam ab8898) and anti-H3K27ac (Active Motif 39133) antibodies, respectively.

### Recombinant protein expression and purification

The gene encompassing the C-terminal array of ZNF506 ZF_1–8_ (residues 201–419) was subcloned into pGEX-6p1. The protein was expressed as a GST-tagged fusion protein in Rosetta 2 (DE3) chemically competent cells. Cells were grown in Luria–Bertani medium at 37°C until the OD_600_ reached 0.6, at which time the temperature was lowered to 16°C. The culture was supplemented with 100 μM ZnCl_2_, and induced by 0.2 mM isopropyl-β-d-1-thiogalactopyranoside (IPTG) overnight. Cells were harvested by centrifugation and resuspended in lysis buffer containing 20 mM Tris pH 7.5, 500 mM NaCl, 5% glycerol, 25 μM ZnCl_2_, 1 mM dl-dithiothreitol (DTT) and 0.1 mM phenylmethylsulfonyl fluoride (PMSF). Cells were lysed and crushed using 1000 MPa of pressure three times and clarified by centrifugation. Clear lysate was loaded onto gravity columns with glutathione beads (SMART) and GST-tagged protein was eluted from the columns with buffer containing 100 mM Tris pH 8, 500 mM NaCl, 25 μM ZnCl_2_ and 10 mM reduced glutathione.

### Isothermal titration calorimetry (ITC) binding assay

ZNF506 ZF_1–8_ protein was diluted to 2.00 × 10^−5^ M and double-stranded DNA probes were diluted to 200 × 10^−9^ M with buffer containing 100 mM Tris pH 8, 500 mM NaCl, 25 μM ZnCl_2_ and 10 mM reduced glutathione. Titration experiments were performed using a MicroCal PEAQ-ITC (Malvern Panalytical) using 20 drops in total, 2 μl per drop over 4 s, with 120 s intervals. *K*_D_ values and curves were analyzed using MicroCal PEAQ-ITC Analysis Software, and the final data were plotted as Δ*H* (kcal/mol) versus the molar ratio.

### Co-immunoprecipitation and western blotting

For co-immunoprecipitation, 293T cells were co-transfected with FLAG-tagged ZNF506-KRAB, HA-tagged SETDB1 and Myc-tagged KAP1 plasmids. After 48 h, cells were pelleted and washed twice with cold phosphate-buffered saline, and proteins were extracted in RIPA buffer or IP buffer [20 mM Tris pH 7.5, 150 mM NaCl, 1% Triton X-100 and a proteinase inhibitor cocktail (Roche)]. Cell extracts were incubated with protein A/G magnetic beads (ThermoFisher) and cross-linked with anti-HA (C29F4 Cell Signaling Technology), anti-FLAG (Sigma F1804) or anti-Myc (BBI) antibodies at 4°C overnight. Beads were then washed three times with BC150 buffer (10 mM Tris pH 7.5, 0.5 mM EDTA, 10% glycerol, 150 mM NaCl and a proteinase inhibitor cocktail). For western blotting, the primary antibodies α-GFP (Invitrogen A11122), α-glyceraldehyde phosphate dehydrogenase (GAPDH) (Proteintech Cat# 60004–1-Ig) and α-red fluorescent protein (RFP) (Abcam ab183628) were used to test specific proteins.

### Virus production and cell infection

Stock pMX-RFP pseudovirus and pBABE-CMV-RFP pseudovirus were generated by co-transfecting 293T cells with 10 μg of pMX-RFP/pBABE-CMV-RFP, 9 μg of pUMVC and 1 μg pMD2.G (Addgene #12259) in 10 cm dishes. The medium was changed 6–8 h after transfection, and the supernatant was collected after 48–72 h. The supernatant was filtered through a 0.45 μm membrane to clear the cell debris, and then collected for infection or concentrated using polyethylene glycol (PEG) 8000 at a 10% final concentration with rolling overnight at 4°C, followed by centrifuging at 3500 *g* for 30 min. 293T cells (1 × 10^5^ cells) were infected with 200 μl of concentrated pMX-RFP pseudovirus and pBABE-CMV-RFP pseudovirus supernatants in 96-well plates. The cells were washed twice with phosphate-buffered saline 4–6 h after infection and transferred to 24-well plates with fresh medium. Cells were harvested 48–72 h after infection for fluorescence-activated cell sorting (FACS).

### Real-time PCR quantitation of viral DNA copies

For viral DNA copy quantitation, genomic DNA from infected cells was harvested and purified using phenol–chloroform 12–48 h post-infection. Early reverse transcripts were quantitated with primers qMXU5-F/R targeting the 5′ LTR U5 of the pMX-RFP pseudovirus. Late reverse transcripts were quantitated with primers specific to the RFP gene in the pMX-RFP vectors. Standard curves were generated with a gradient dilution of pMX-RFP vectors for each primer. qPCR was performed with 100 ng of cell DNA using MagicSYBR mixture (CWBio CW3008). An Alu-based two-round nested PCR was performed to quantitate integrated viral DNA. PCR mixtures containing 100 ng DNA samples, the primers LambdaT-MX, Alu1 and Alu2, and Super TaqMan mixture (CWBio CW2698) were used. The PCR cycle conditions were 2 min at 95°C, followed by 20 cycles of 15 s at 95°C and 10 s at 60°C, and 3 min at 72°C. PCR products were diluted at 1:10 for a second nested real-time PCR using the primers Lambda T and Nested-R, the probe MXU and Super Taqman mixture.

### Evolutionary analysis

We extracted seven orthologs of ZNF506 from the NCBI database (https://www.ncbi.nlm.nih.gov/gene), and the ratios of non-synonymous substitutions to synonymous substitutions (dN/dS) between the ortholog proteins were calculated using MEGA-X v10.1.8 (41). The KZFP sequences and annotations were downloaded from the UniProt database. MEGA-X v10.1.8 (41) was used to produce multiple sequence alignments and construct maximum likelihood trees. FigTree v1.4.4 (github.com/rambaut/figtree) was used to plot phylogenetic trees. KZFP pairwise sequence alignments were produced using EMBOSS Needle (42), and the matrix is BLOSUM62. Comparisons of candidate KZFPs in different species were plotted by using TreeAndLeaf (43).

### The relationship between the expression level of KZFPs and various cancers

We used gene expression data in GEPIA (Gene Expression Profiling Interactive Analysis; http://gepia.cancer-pku.cn) to explore the link between KZFPs and various cancers. For KZFPs, those KZFPs whose expression was doubled and had a *P*-value < 0.01 were considered to have significantly increased expression. We counted the number of up-regulated KZFPs in all cancers presented by GEPIA. AML is one of the cancer types with the most significant up-regulation of KZFPs.

We further analyzed these up-regulated KZFPs in AML; the expression of ZNF506 was significantly up-regulated, and the up-regulation fold ranked second.

### Relationship between disease-free survival rate and expression of KZFPs in AML patients

AML patients were categorized into two groups based on their level of KZFP expression [log2(FC) >2 and *P*-value < 0.01]. Using tools provided by GEPIA, we analyzed and plotted the difference in disease-free survival between these two groups of patients.

## RESULTS

### Genome-wide mapping of ZNF506-binding sites shows enrichment at PBS-pro sites of ERVP subfamilies

**Figure 1.**
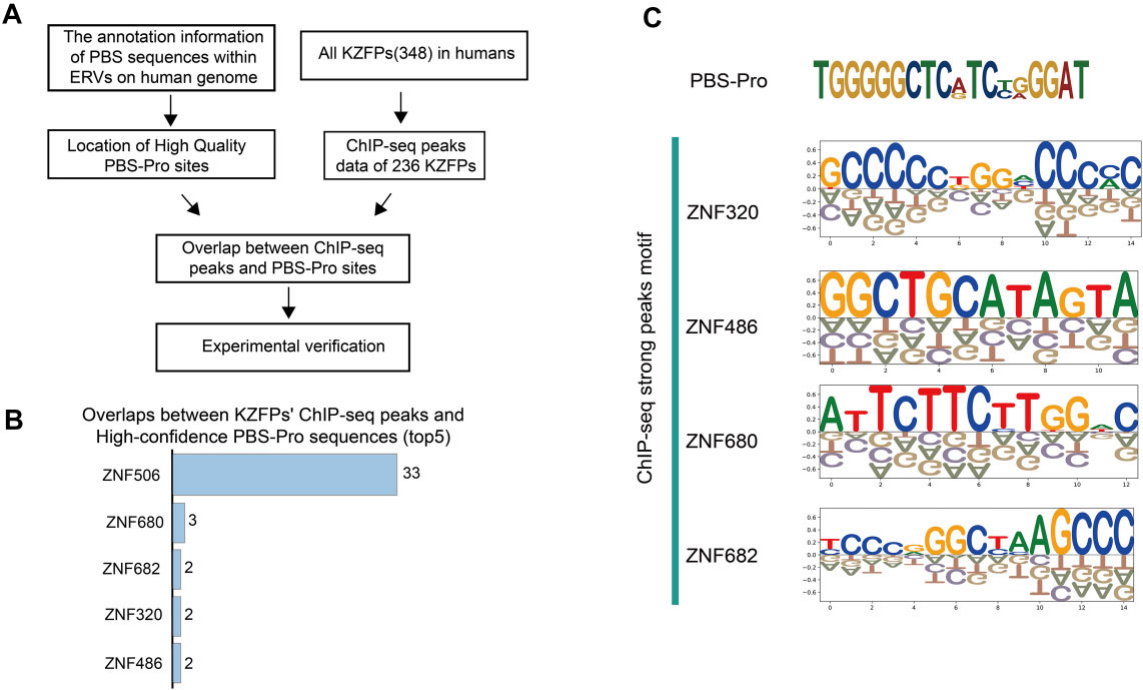
Screening KZFPs targeting PBS-Pro in humans. (**A**) Pipeline for screening KZFPs targeting PBS-Pro in humans. The ChIP-seq data of KZFPs mainly come from the Cistrome Data Browser, with most of the raw data derived from the study conducted by Imbeault *et al*. (16) (GEO: GSE78099). (**B**) The top five KZFPs with overlapping ChIP-seq peaks in PBS-Pro sites, along with their corresponding counts. PBS-Pro sequences were chosen based on RetroTector scores, with records scoring <100 excluded. (**C**) DNA-binding motifs of the other four KZFPs as presented in (B).

To identify KZFPs capable of binding to PBS-Pro sequences in humans, we utilized an effective strategy for possible KZFP screening (Figure 1A). Specifically, we employed the RetroTector software (35) for a comprehensive investigation of intact ERVs within the human genome, from which we obtained their PBS types and corresponding confidence scores. To validate the accuracy of PBS classification by the software, we followed the previously established approach (24), opting for high-confidence PBS-Pro candidates (score > 100). As a result, 46 credible PBS-Pro annotations were identified in the human genome (Supplementary Table S1).

Next, we integrated the public ChIP-seq data of human KZFPs (16,36) by analyzing their peaks in relation to the calculated PBS-Pro regions. Among the 236 KZFPs with available ChIP-seq experimental data, ZNF506 demonstrated significant binding affinity towards PBS-Pro sequences (Figure 1B; Supplementary Table S1). Additionally, another four KZFPs with few overlaps of ChIP-seq peaks and PBS-Pro sites also displayed distinctively different binding motifs compared with PBS-Pro sequences (Figure 1C).

To date, there are 348 distinct KZFPs identified in the human genome. To discover additional KZFPs with potential PBS-Pro binding activity among the remaining 112 KZFPs lacking ChIP-seq data, we utilized an evolutionary and predictive approach to assess the binding relationship between these KZFPs and ERVPs (Supplementary Figure S1a, b; Supplementary Table S1). Despite these efforts, we failed to identify any promising candidates. Additionally, when juxtaposing the fingerprint profiles of these 112 KZFPs with those of ZNF506 and ZFP809, we observed a low degree of similarity (<50%, Supplementary Figure S1c). These results indicate that the interaction between ZNF506 and PBS-Pro sequences might be unique among human KZFPs.

**Figure 2.**
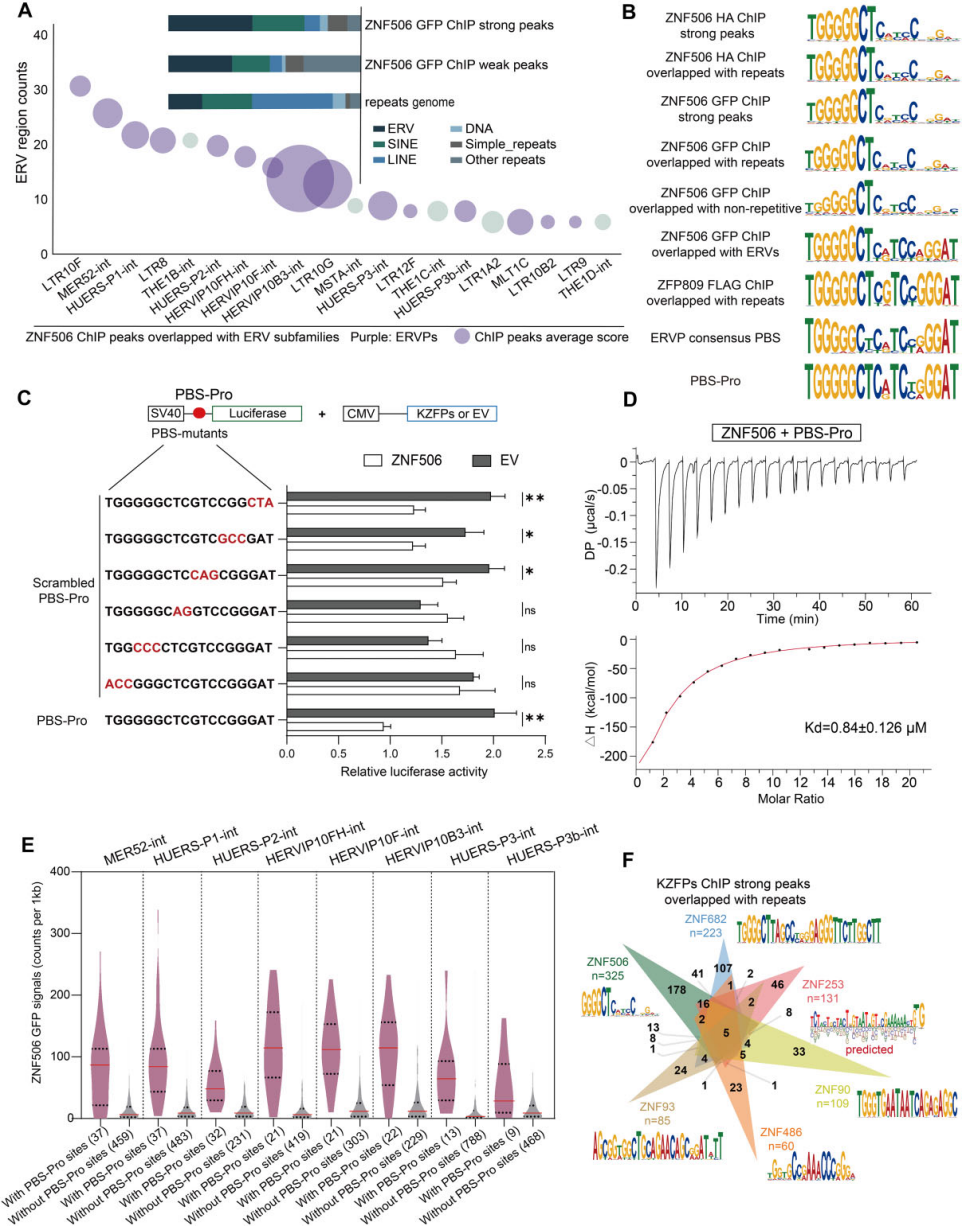
Genome-wide mapping of ZNF506-binding sites shows enrichment at ERVP subfamilies. (**A**) Strong ZNF506–GFP ChIP-seq peaks overlapped with repeat regions and the human repeat genome as determined by genomic overlap with RepeatMasker annotations (top). Plot showing ZNF506 strong peak numbers and scores at ERV regions (bottom). ERVP subfamilies are marked in purple. Bubbles are sized according to average scores of ChIP-seq peaks. The cell line used is 293T. (**B**) ChIP-seq motifs of ZNF506 compared with the PBS-Pro consensus sequences. Consensus ZNF506 target motifs were analyzed from the high confidence peaks (peak score > 500). HA-tagged ZNF506 ChIP-seq data were from Imbeault *et al.* (16) (SRR accession: SRR5197160). ZFP809 ChIP-seq data were from Wolf *et al.* (28) (SRA accession: SRR1188176). (**C**) Relative luciferase activity of 293T cells overexpressing ZNF506 or an empty vector (EV) and SV40 promoter-driven reporter containing the PBS-Pro sequence with the indicated mutations highlighted in red (left). *t*-test: error bars indicate the standard deviation (SD); *P < 0.05, **P < 0.01, ns *P* > 0.05; *n* = 3. (**D**) ITC binding assay of PBS-Pro double-stranded oligonucleotides titrated with ZNF506-ZF_1–8_ protein. (**E**) Violin plot showing ZNF506–GFP signals at ERVP subfamilies with or without PBS-Pro sites. Means are marked in red, and quartiles are represented by dashed lines. (**F**) Venn diagram showing the overlap among strong ZNF506, ZNF253, ZNF682, ZNF93, ZNF90 and ZNF486 ChIP-seq peaks at repeat regions, with the ChIP-seq motif indicated.

To validate the binding between ZNF506 and PBS-Pro sequences experimentally, a transposon vector-based ZNF506–GFP was stably transfected in 293T cells (Supplementary Figure S2a, b), which were then subjected to ChIP-seq using GFP antibodies. We integrated the published ZNF506 HA ChIP-seq data (SRR5197160) using HA antibodies with our ChIP-seq data for peak analysis. Most of the binding peaks of both the published and our ChIP-seq results were located in repeat regions of the genome with a high degree of coincidence, especially the strong peaks (GFP ChIP-seq peak score > 500, and HA ChIP-seq peak score > 300 using MACS analysis software) (Supplementary Figure S2c, d). Among the repetitive regions, we detected strong enrichment of ZNF506 binding peaks located at ERV regions that possess PBS-Pro sequences, including HUERS-P1-int, HUERS-P2-int and MER52-int (Figure 2A; Supplementary Figure S2e).

Consistent with the ZNF506 HA ChIP-seq data, motif calling analysis of our ChIP-seq data revealed that ZNF506 bound to a PBS-Pro-like motif, especially the first nine nucleotides that showed high similarities with ERVP PBS consensus sequences (Figure 2B). Interestingly, unlike ZFP809 specifically bound to only one kind of PBS-Pro sequence in mice, as its binding capacity was dramatically decreased once any base of the PBS-Pro sequences was mutated (10,16,44), ZNF506 showed higher tolerance in the targeting sequences and was capable of binding to readily mutated PBS-Pro sequences of different ERVP subfamilies, which may be related to the lower binding score of the last nine nucleotides in the target motif of ZNF506 (Figure 2B). We compared the consensus sequences of these ZNF506-binding ERVP families within the first 50 base pairs. The results showed that TGGGGGCTC, the first nine nucleotides of the consensus sequences, are highly conserved among most of the ERVPs, while other regions are less conserved (Supplementary Figure S2f). Therefore, ZNF506 can accurately bind to these ERVPs by recognizing the first nine nucleotides to play a repressive role. We further investigated several major types of PBS sequences in human ERVs and found that PBS-Pro differs significantly from other types of PBS sequences within the first nine nucleotides (Supplementary Figure S2g), indicating that ZNF506 can specifically bind to PBS-Pro.

To verify the binding motifs of ZNF506, we performed luciferase reporter assays using an SV40 promoter-driven luciferase plasmid containing different PBS sequences (such as PBS-Lys and PBS-Phe) (Supplementary Figure S2h). While expression of ZNF506 significantly repressed PBS-Pro-containing luciferase activity, constructs of other types of PBS sequences abolished the ZNF506 repression activity, suggesting its binding specificity for the PBS-Pro sequences. We also found that triple scrambling of the PBS-Pro sequences affected the extent of transcriptional repression of ZNF506 differently. Triple scrambling of the first nine nucleotides of the PBS-Pro sequences diminished the repression activity of ZNF506, while mutations in the last nine nucleotides had little impact on transcriptional repression (Figure 2C), indicating that ZNF506 had different binding capacities to nucleotides of the PBS-Pro consensus sequences, consistent with the motif analysis results (Figure 2B).

To further validate ZNF506 binding to the PBS-Pro sequences, we ectopically expressed the C-terminus of ZNF506 in *Escherichia coli*, purified the eight ZFs of ZNF506 (Supplementary Figure S2i) and measured the binding affinity for a 28 bp double-stranded oligonucleotide encompassing PBS-Pro sequences using ITC. We found that the ZFs of ZNF506 potently bound to the PBS-Pro sequence with a *K*_D_ = 0.84 ± 0.126 μM (Figure 2D; Supplementary Figure S2j). To analyze whether the enrichment of ZNF506 on the ERVP loci was due to its targeting of PBS-Pro sequences, we categorized different ERVP subfamilies by whether they contained the PBS-Pro sequences. ZNF506 binding capacities displayed significant differences between the two categories of ERVP groups (Figure 2E; Supplementary Figure S3a). Taken together, genome-wide mapping of ZNF506-binding sites showed strong enrichment at ERVP subfamilies by targeting PBS-Pro sites.

In addition, we analyzed the public ChIP-seq data of ZNF253 (SRR14103920), ZNF93 (SRR5197268), ZNF682 (SRR5197216), ZNF90 (SRR5197267), ZNF486 (SRR5197156), ZNF737 (SRR5197228) and ZNF626 (SRR5197202), all of which are located close to the *ZNF506* gene locus in the same cluster on chromosome 19. Analysis of high confidence peaks of the KZFPs ChIP-seq data indicated their strong enrichment in repeat regions (Supplementary Figure S3b). However, none of them displayed significant binding capacities to the targeting regions of ZNF506 or PBS-Pro sites (Figure 2F; Supplementary Figure S3c, d). ZNF253 inhibited the transcriptional activities of AP-1 and SRE (45), ZNF93 bound to L1 retrotransposons (18), ZNF682 bound to MER66 LTR regions, ZNF90 bound to HERVH-int subfamilies and ZNF486 bound to PBS-Phe sites (30) (Supplementary Figure S3e, f), with even these KZFPs in the same cluster showing high similarity and similar binding motifs to ZNF506 (Supplementary Figure S3e). We noticed that ZNF765 also targeted the THE1 subfamilies with a binding motif similar to ZNF506, particularly at the first nine nucleotides (Supplementary Figure S3e), while showing distinct preferences in binding sites (Supplementary Figure S3g). Thus, even though KZFPs in the same cluster on a chromosome probably have similarities in protein sequences and binding motifs, they have developed to locate their target sites to perform independent functions.

Unlike ZNF506 that specifically recognized the first nine nucleotides of the PBS-Pro sequences (Figure 2B, C), ZFP809 targeted the entire 18 nucleotides of the PBS-Pro sequences since mutations of either the first or last nine nucleotides of the PBS-Pro sequences significantly abolished its repression capacity in luciferase reporter assays (Supplementary Figure S4a), consistent with the binding motif (Figure 2B) (28) and electrophoresis mobility shift assay results (10). We also observed stronger repression of transcription by ZFP809 than by ZNF506 (Supplementary Figure S4a). We constructed various KZFP mutant plasmids by exchanging their KRAB domains, ZF arrays or the linkers connecting the KRAB domain and ZF array, and performed luciferase reporter assays. Interestingly, ZNF506 mutants with the ZFP809 ZF array showed increased repression activities that were comparable with ZFP809. In contrast, ZFP809 mutants using the ZNF506 ZF array exerted similar repression capacities to ZNF506, which were lower than that of the ZFP809 wild-type (WT) protein (Supplementary Figure S4b), indicating that differences in transcriptional repression may be due to the differences in the ZF arrays of these two KZFPs.

Collectively, our genome-wide mapping results showed that ZNF506 interacted with PBS-Pro sequences specifically for transcriptional repression of the targeted ERVP subfamilies.

### ZNF506 recruits co-repressors to promote the formation of H3K9me3 for transcriptional repression

**Figure 3.**
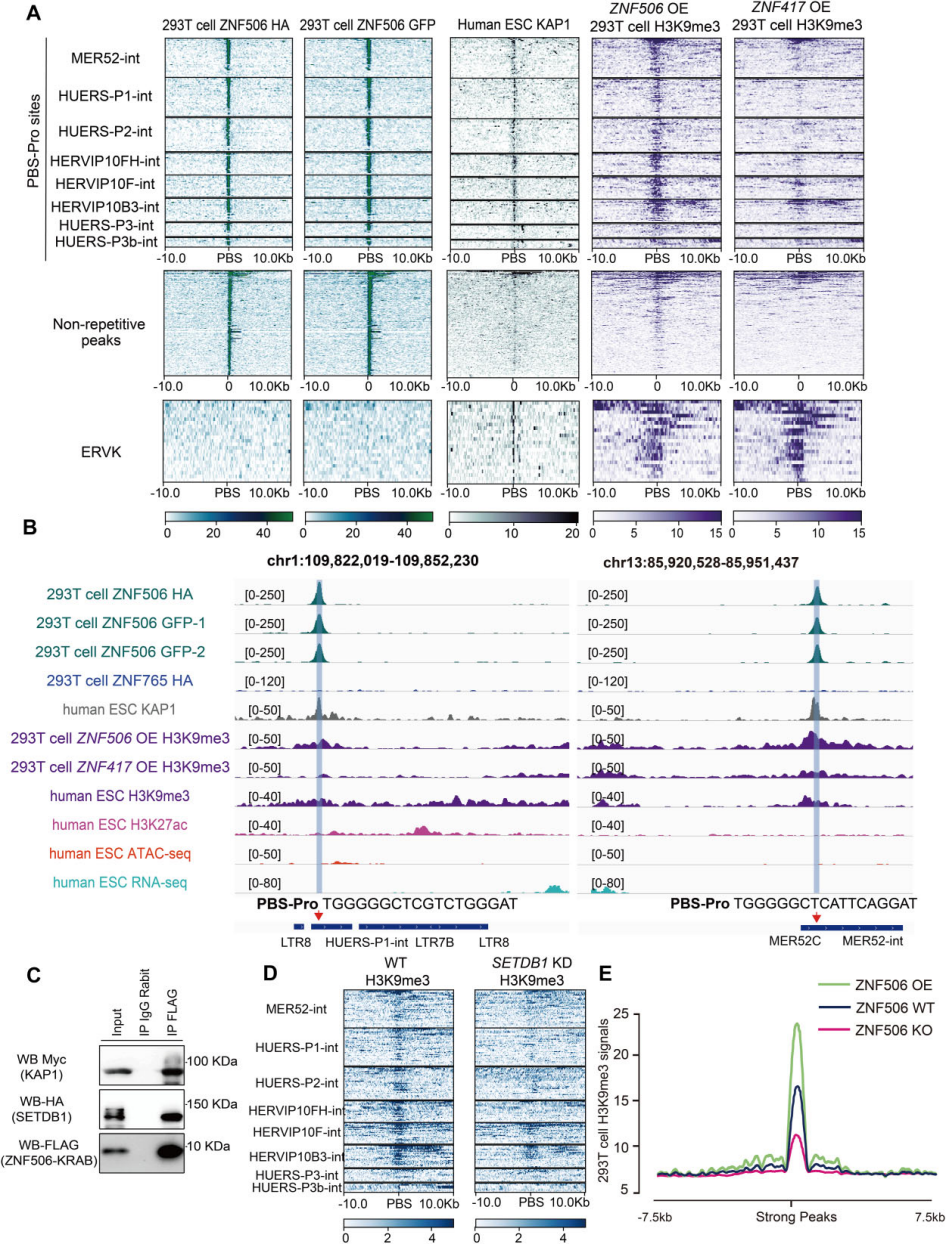
ZNF506 recruits KAP1 and SETDB1 to promote the formation of H3K9me3 modifications. (**A**) Heatmap showing ZNF506, KAP1 and H3K9me3 signals at ERV-associated PBS-Pro sites and at strong non-repetitive ZNF506 ChIP-seq peak regions. The 20 kb regions are displayed with the PBS-Pro sequences or with non-repetitive peaks positioned at the center of the peak regions. OE, overexpression. (**B**) ChIP-seq signals of ZNF506, ZNF765, KAP1, H3K9me3, H3K27ac, ATAC-seq and RNA-seq at ERVP subfamilies containing PBS-Pro sequences. (**C**) Co-immunoprecipitation and western blotting for the FLAG-ZNF506 KRAB domain with Myc-KAP1 and HA-SETDB1. (**D**) Heatmap showing H3K9me3 signals at ERVP subfamilies with PBS-Pro sites before and after SETDB1 knockdown (KD) in a melanoma cell line. (**E**) Profiles showing H3K9me3 signals at different distances from ZNF506 strong peaks in *ZNF506* OE, *ZNF506* WT and *ZNF506* KO 293T cells.

We next tested the co-occupancy of ZNF506, KAP1 (SRR3178875) and H3K9me3 signals at sites in repeats or non-repetitive regions in cells with *ZNF506* overexpression (OE). Similar to other classical KZFPs (28,30,46), ZNF506 binding was positively correlated with KAP1 in repeat regions compared with non-repetitive regions (Figure 3A). We also observed enrichment of H3K9me3 signals at ZNF506/KAP1 co-occupied sites in ERVP subfamilies containing PBS-Pro sites, compared with H3K9me3 signals in samples overexpressing *ZNF417*, a recently identified PBS-Lys-binding protein (30,31) (Figure 3A). These data collectively suggested that ZNF506 may function via KAP1 to produce H3K9me3 enrichment at ERVP subfamilies by targeting the PBS-Pro sites. Interestingly, by analyzing the public sequencing data of human embryonic stem cells (hESCs) (SRR8983692, SRR14005187 and SRR14005189), we observed significant enrichment of H3K9me3 repressive modifications and a mild increase in the enrichment of DNA methyltransferase (DNMT3A and DNMT3B) at PBS-Pro-containing ERVP subfamilies in hESCs, compared with ERVP subfamilies without PBS-Pro sites (Supplementary Figure S5a).

ZNF506 bound to the PBS-Pro sites of ERVP subfamilies and recruited KAP1, resulting in heterochromatization of nearby regions by H3K9me3 modifications (Figure 3B). We also observed a lack of enrichment of active H3K27ac histone modifications in ZNF506-binding regions, as well as transcriptional repression of the targeted repeats (Figure 3B). In addition, the binding capacities of ZNF506 were lost when mutations were present in the first nine nucleotides of the PBS-Pro sequences of ERVP subfamilies (Supplementary Figure S5b), consistent with our observations in luciferase assays. However, ZNF506 also bound to non-ERVP subfamilies with PBS elements mutated into PBS-Pro sequences, leading to recruitment of KAP1 as well as enrichment of H3K9me3 signals (Supplementary Figure S5b). We also confirmed the different binding preferences between ZNF506 and ZNF765 (Supplementary Figure S3g). The two KZFPs bound to distinct THE1 subfamilies and recruited KAP1 for H3K9me3 modifications (Supplementary Figure S5c).

We confirmed the physical interaction between ZNF506 and the potential co-repressors KAP1 and SETDB1 by co-immunoprecipitation in 293T cells overexpressing the FLAG-tagged ZNF506-KRAB domain (amino acids 1–75) with Myc-tagged KAP1 or HA-tagged SETDB1 with α-FLAG, α-Myc or α-HA antibodies, respectively (Figure 3C). Furthermore, knockdown of *SETDB1* resulted in clearly decreased H3K9me3 signals and slightly increased H3K4me1 modifications in ZNF506-binding ERV regions (Supplementary Figure S6a, b), particularly the PBS-Pro-containing ERVP subfamilies (47) (Figure 3D), while both H3K9me3 and H3K4me1 signals on the ERVP subfamilies without PBS-Pro sites were not affected (Supplementary Figure S6c, d).

To further explore H3K9me3 patterns in ZNF506-binding regions, we generated *ZNF506* KO 293T cell lines by using CRISPR/Cas9 [clustered regularly interspaced palindromic repeats (CRISPR)/CRISPR-associated protein 9] engineering (Supplementary Figure S7a, b) and performed ChIP-seq experiments on *ZNF506* OE, *ZNF506* WT and *ZNF506* KO cells. Calculating the densities of ChIP-seq reads at different distances from ZNF506-binding sites by using Deeptools (48), we found different levels of H3K9me3 signals close to the ZNF506 peak regions among *ZNF506* OE, *ZNF506* WT and *ZNF506* KO cells (Figure 3E). In addition, the difference in H3K9me3 enrichment was mainly observed in PBS-Pro sites such as HUERS-P2 and HERVIP10FH subfamilies, rather than PBS-Lys-containing regions (Supplementary Figure S7c, d). Taken together, these results indicated that ZNF506 functions as a heterochromatic repressor in combination with the co-repressors KAP1 and SETDB1, leading to H3K9me3 enrichment in ERVP subfamilies by targeting the PBS-Pro sequences.

### ZNF506 is a potential repressor of PBS-Pro-utilizing pseudoviral infection

**Figure 4.**
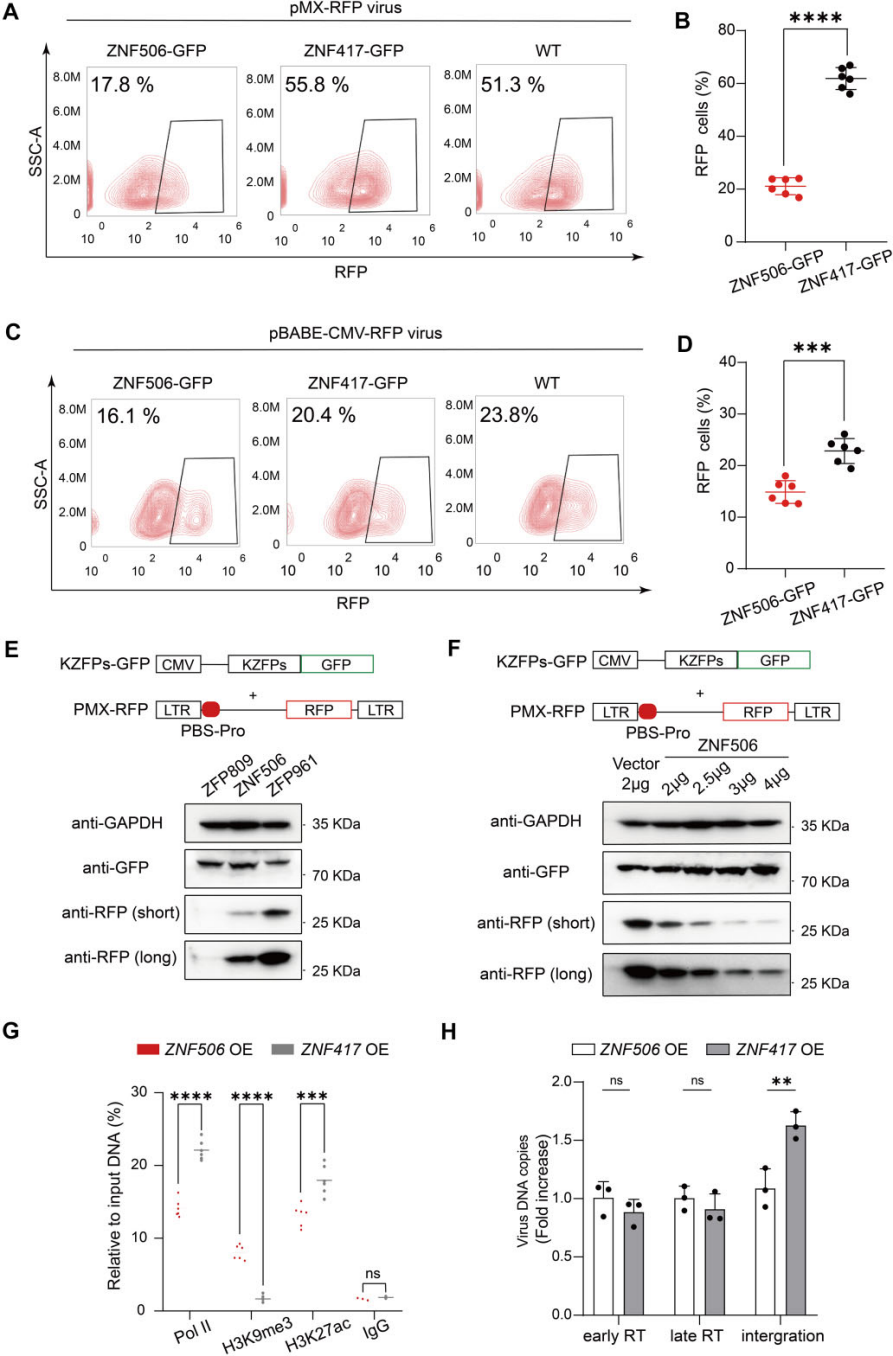
ZNF506 restricts PBS-Pro-utilizing pseudoviral infectivity. (A and B) FACS analysis (**A**) and statistical plot (**B**) showing the pMX-RFP viral infection rate in 293T cells stably overexpressing GFP-tagged *ZNF506* or *ZNF417*, or WT cells. *t*-test: error bars indicate the SD; ****P < 0.0001, *n* = 6. (C and D) FACS analysis (**C**) and statistical plot (**D**) showing the pBABE-CMV-RFP viral infection rate in 293T cells stably overexpressing GFP-tagged *ZNF506* or *ZNF417*, or WT cells. *t*-test: error bars indicate the SD; ***P < 0.001, *n* = 6. (**E**) Western blot showing expression of RFP in 293T cells co-transfected with the pMX-RFP vector and ZFP809, ZNF506 vectors, or ZFP961 as a control. (**F**) Western blot showing expression of RFP in 293T cells co-transfected with the pMX-RFP vector and different amounts of ZNF506 plasmids. (**G**) ChIP assays with antibodies against Pol II, H3K9me3 and H3K27ac and an immunoglobulin G (IgG) control in 293T cells overexpressing GFP-tagged *ZNF506*, or *ZNF417* as a control, followed by qPCR using primers specific for the pMX LTR. *t-*test: error bars indicate the SD; ***P < 0.001, ****P < 0.0001, ns *P* > 0.05; *n* = 3 or 6. OE, overexpression. (**H**) Histogram showing viral DNA copy numbers in 293T cells overexpressing GFP-tagged *ZNF506*, or *ZNF417* as a control. *t*-test: error bars indicate the SD; **P < 0.01, ns *P* > 0.05; *n* = 3. RT, reverse transcription.

As ZNF506 specifically targets PBS-Pro sequences for transcriptional repression, we next tested whether ZNF506 may also influence PBS-Pro-utilizing viral infectivity (Supplementary Figure S8a, b). We generated pseudoviral particles based on pBABE-CMV-RFP or pMX-RFP plasmids, both containing a PBS-Pro element (Supplementary Figure S8b). The major difference between the two viral plasmids was that RFP expression was driven by the transcriptional activity of the LTR promoter in pMX-RFP, while RFP was constitutively expressed under the control of the CMV promoter in pBABE-CMV-RFP, indicating the integration efficiency of the retrovirus (49). The viral particles were infected into ZNF506–GFP 293T cells, and ZNF417–GFP cells were used as a control. FACS analysis determining the ratios of RFP^+^ cells in GFP^+^ cells revealed that *ZNF506* overexpression significantly reduced pMX-RFP pseudoviral infectivity, compared with WT cells or control cells overexpressing *ZNF417* (Figure 4A, B), but showed much less repression of pBABE-RFP pseudoviral infection (Figure 4C, D). We also performed viral infection experiments in 293T cells transfected with GFP-fused KZFPs, including the PBS-Pro-binding human ZNF506 and mouse ZFP809 proteins, and the PBS-Lys-binding human ZNF417 and mouse ZFP961 proteins. We observed significantly reduced pseudoviral infectivity in *ZNF506* and *Zfp809* OE cells, compared with that in *ZNF417* and *Zfp961* OE cells (Supplementary Figure S8c, d). Thus, these results suggested that ZNF506 could restrict the infectivity of pseudoviruses containing the PBS-Pro sequences.

### ZNF506 inhibits viral infection by transcriptional repression of the integrated viral DNA

After invading host cells, exogenous retroviruses undergo a series of events: uncoating; reverse transcription of their RNA into proviral DNA by recruiting host-specific tRNA to the PBSs; integration of the double-stranded DNA into the host genome; and, finally, transcription into RNA for viral protein expression by co-opting the host machinery (25,50). As ZNF506 can potently repress the infectivity of PBS-Pro-utilizing viruses, we next tested which step of viral infection ZNF506 affected. We co-transfected pMX-RFP pseudoviral plasmids with GFP-fused KZFP vectors into 293T cells. FACS and western blotting results showed that the RFP expression was dramatically repressed by ZNF506 or ZFP809 compared with ZFP961 as a control (Figure 4E; Supplementary Figure S8e, f). Moreover, RFP expression also gradually decreased when ZNF506 or ZFP809 expression increased by transfection; however, this result was not observed in the ZFP961 expression group (Figure 4F; Supplementary Figure S8g). In addition, we also observed stronger repression of RFP expression by ZFP809 than by ZNF506 (Figure 4E; Supplementary Figure S8e), consistent with the luciferase assay results (Supplementary Figure S4a). We also found that the rescued expression of ZNF506 co-transfected with the pMX-RFP vector in *ZNF506* KO cells restored the repression of RFP, while ZNF417 or ZFP961 could not (Supplementary Figure S8h), further suggesting the ability of ZNF506 to inhibit PBS-Pro-utilizing pseudoviral infection.

We then performed ChIP followed by qPCR experiments using ZNF506–GFP and ZNF417–GFP 293T cells infected with the pMX-RFP pseudoviruses, using antibodies against Pol II, H3K9me3 and H3K27ac. qPCR results showed a significant decrease in Pol II recruitment and H3K27ac signals, as well as a dramatic increase of H3K9me3 levels at the viral LTR regions in *ZNF506* OE cells, compared with the levels in *ZNF417* OE cells (Figure 4G). Finally, we calculated the copy numbers of the pMX-RFP pseudoviruses in infected ZNF506–GFP and ZNF417–GFP 293T cells at different stages, including 12 h after infection to test early reverse transcription (early RT), 24 h after infection to test late reverse transcription (late RT) and 48 h after infection to test viral integration into the genome (51). qPCR results revealed that *ZNF506* OE affected viral integration slightly but showed no repression ability in the early or late RT processes (Figure 4H), suggesting that ZNF506 repressed PBS-Pro-utilizing pseudoviruses mainly by transcriptional repression of the integrated viral DNA.

### ZNF506 evolves under constraints in the primate lineage

**Figure 5.**
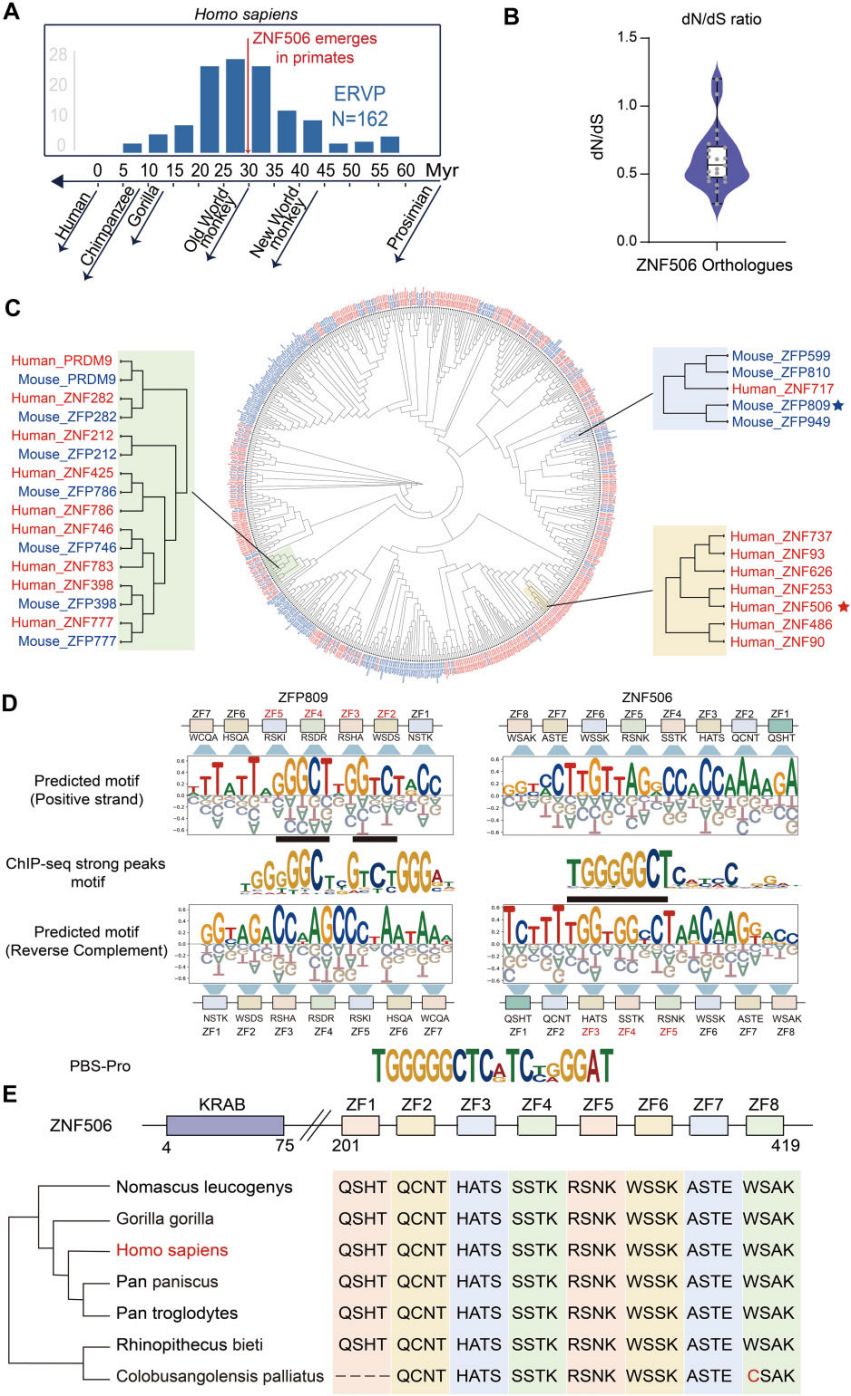
ZNF506 evolves independently in humans against ERVP. (**A**) Histogram showing the timing of genome insertion of PBS-Pro-containing ERV subfamilies during primate evolution. Myr, million years. (**B**) Violin plot showing dN/dS ratios of ZNF506 orthologs in primates. (**C)** Phylogenetic tree showing the evolutionary relationship of KZFPs in humans and mice according to similarities of the KRAB domains. Larger images show details on ancient and homologous KZFPs between mice and humans (green block), KZFPs with high similarities to ZNF506 (yellow block) and KZFPs with high similarities to ZFP809 (blue block). Mouse KZFPs are in blue, and human KZFPs are in red. (**D**) Alignment of DNA-binding motifs of ZNF506 and ZFP809 between the predicted motif and ChIP motif. The correspondence between ZFs and bases is represented by blue trapezoids. The overlap between the predicted motif and the ChIP motif is indicated by a black rectangle. The fingerprint of each zinc finger is shown below. The ChIP-seq motif of ZFP809 matched the positive strand of the predicted result, while ZNF506 matched the reverse complementary strand of the predicted result. Their ZFs that are critical for binding are also different. High confidence peaks (peak score > 500 or fold change > 50) are used to extract the ChIP-seq motif for either ZNF506 or ZFP809. (**E**) Domain structure of ZNF506 (top). Multi-sequence alignments of DNA-contacting residues in the ZF array of selected ZNF506 orthologs (bottom). The four key amino acids shown per ZF are defined at positions −1, 2, 3 and 6 relative to the first histidine of each C2H2 ZF.

Previous studies have provided evidence of the co-evolution between KZFPs and ERVs (52). To investigate the potential evolutionary relationship between ZNF506 and the ERVP subfamily within the human genome, we conducted an analysis of the LTR sequences. Our findings indicate that the ERVP subfamilies primarily appeared in the genome of Old World monkeys ∼30 million years ago (Figure 5A). Using the most recent common ancestor (MRCA), we compared the emergence of ZNF506 in the primate genome with the time of ERVP invasion, and determined that they occurred concurrently (Supplementary Table S1). Despite being a rapidly evolving protein, ZNF506 was found to be conserved in primates (Figure 5B). This correlation between KZFPs and ERVs throughout evolutionary time highlights the crucial role of ZNF506 in regulating ERVP invasion and maintaining genome stability.

Despite their shared function of binding to PBS-Pro, ZFP809 and ZNF506 exhibit relatively low similarities in protein homology and fingerprint amino acids. Comparing the amino acid sequences between ZNF506 and ZFP809 showed that they shared a similarity of only 46.5%, with 66.7% in the KRAB domain and 60.5% in the ZF array. These percentages of homology are in line with random expectations for comparisons of any two KZFPs (Supplementary Figure S9a). The phylogenetic tree also confirmed the absence of an evolutionary relationship between the two KZFPs, differing from the ancient KZFPs with homology between humans and mice, such as PRDM9 and ZNF746 (Figure 5C). Moreover, the four fingerprint amino acids in the ZF arrays were also fundamentally different between ZNF506 and ZFP809 (Supplementary Figure S9b).

Next, we combined the experimental and predicted results to analyze the ZF binding characteristics between ZNF506 and ZFP809. Despite both binding to PBS-Pro, ZNF506 and ZFP809 have different preferences for different parts of PBS-Pro (Figure 5D), which can be attributed to the distinct ZFs on the tandem ZF domain responsible for base recognition and binding. Specifically, ZNF506 exhibits a preference towards binding to the first nine nucleotides within PBS-Pro. Conversely, ZFP809 is capable of recognizing the entire PBS-Pro sequences, yet demonstrates a greater propensity for binding to the central two fragments (Figure 5D). The third to fifth ZFs of ZNF506 are particularly influential in this process, while the second to fifth ZFs of ZFP809 may be involved (Figure 5D). Previous studies have also pointed out the key role of ZF3, 4 and 5 of ZFP809 for PBS-Pro binding (26). Nevertheless, the impact on DNA binding specificity by other ZFs within these two KZFPs remains challenging to delineate.

The difference in the binding of ZNF506 and ZFP809 to the positive and negative strands of DNA may also be one reason for the low similarity of their ZFs (Figure 5D). These results indicate that the two KZFPs may have evolved independently to defend against PBS-Pro-utilizing exogenous retroviruses that invaded the genomes of different ancestors of mice and humans, coinciding with the emergence of ERVP subfamilies.

Interestingly, we found that the KZFPs on the same phylogenetic branch as ZNF506, such as ZNF253 and ZNF90 (Figure 5C), were also located close to the ZNF506 gene locus, within a rapidly evolving KZFP gene cluster on chromosome 19 of the human genome (Supplementary Figure S9c). Among them, ZNF253 was located closest to ZNF506, displaying a 98.6% similarity in amino acid sequences of their KRAB domains (Supplementary Figure S9a). While they share several similar ZFs, they do not possess the same DNA binding specificities (Supplementary Figure S9d). As ZNF506 appeared earlier than ZNF253 in the primate genomes, ZNF253 might have developed from ZNF506 by genetic duplication. Likewise, ZNF506 could also be generated from the older ZNF90 by replication and recombination in the primate genomes during evolution.

Further analysis of ZNF506 proteins across different species of primates showed few mutations in the four key amino acids of their ZF arrays (–1, 2, 3 and 6), which are crucial for recognizing and binding to specific DNA sequences (1,13) (Figure 5E). Additionally, the full ZF and inter-ZF linker regions of these orthologs also exhibited strong homology (Supplementary Figure S9e).

In addition to primates, we also attempted to determine the timing of the emergence of ERVP subfamilies in the genomes of other species during evolution, including *O. aries*, *A. melanoleuca*, *B. musculus*, *M. lucifugus* and *P. leo*. Our analysis confirmed the presence of ERVP subfamilies in these genomes (Supplementary Figure S10a), suggesting that these species may have developed the corresponding KZFPs to target PBS-Pro sequences for defense. To identify the candidate KZFPs, we selected those that emerged simultaneously with PBS-Pro elements (Supplementary Figure S10b–d; Supplementary Table S2). Although this approach may not precisely predict the PBS-Pro-binding KZFPs in these species, it can narrow down the scope to identify the most promising ones.

We also searched for homologous proteins to ZNF506 or ZFP809 in these species but did not find KZFPs possessing >50% similarities in fingerprints to the two KZFPs (Supplementary Figure S11). In fact, when we compared the fingerprint similarity of all the KZFPs in these species with ZNF506 and ZFP809 to exclude any potential omissions, we also failed to find similar KZFPs. These results suggested that genomes of species may be invaded by PBS-Pro-utilizing exogenous retroviruses after species divergence, resulting in the independent evolution of corresponding KZFPs with no homology.

### ZNF506 and other KZFPs may play a role in leukemia

**Figure 6.**
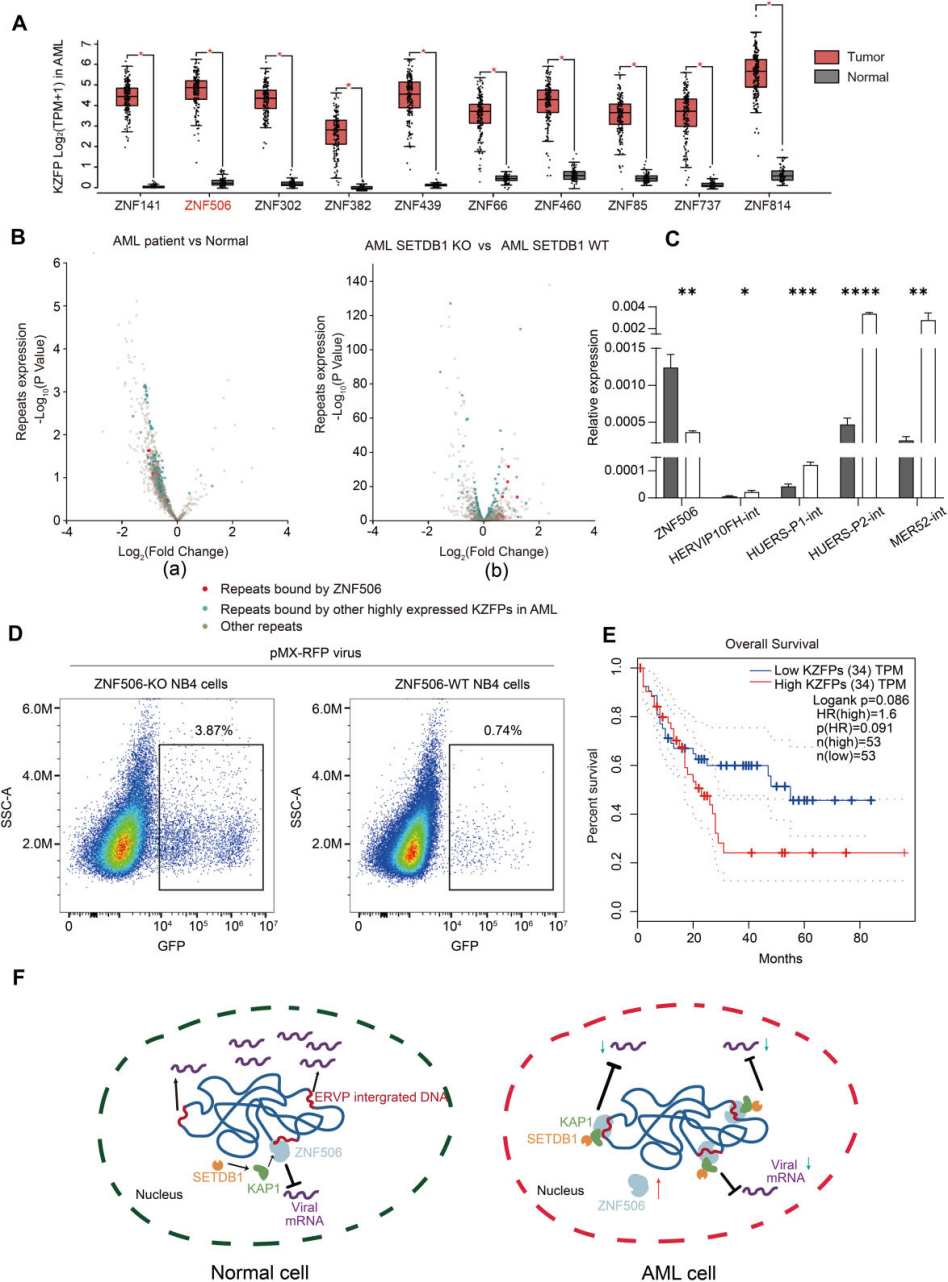
ZNF506 and other KZFPs may play a role in leukemia. (**A**) Top 10 KZFPs significantly up-regulated in AML compared with normal tissue. The KZFPs are ranked by the significance of up-regulation (*P < 0.05, 173 samples from AML patients and 70 samples from healthy individuals), and ZNF506 ranks second among them. (**B**) (a) Changes in various subfamilies of repeats in AML, with ZNF506-bound ERVs marked as red dots (GEO database accession: GSE175701). (b) Changes in the expression of each ERV in AML cells after SETDB1 KO (GEO database accession: GSE103409). (**C**) Expression levels of ZNF506-bound ERVs before and after *ZNF506* KO in NB4 cells. *P < 0.05, **P < 0.01, ***P < 0.001, ****P < 0.0001; *n* = 3. (**D**) FACS analysis showing the viral infection rate of pMX-IRES-GFP in WT and *ZNF506* KO NB4 cells. (**E**) Disease-free survival of high and low KZFP expression groups. Compared with the high expression group, patients in the low expression group of KZFPs had better disease-free survival. (**F**) Schematic diagram illustrating that in AML patients, abnormal KZFP expression disrupts the balance of repeat element expression. As an illustration, the inhibition of ERVP by ZNF506 is utilized as a paradigmatic case.

Next, we tried to investigate the potential association between ZNF506 and diseases. Utilizing the GEPIA (53), we observed aberrant expression of ZNF506 in various cancers, such as thymoma and uterine carcinosarcoma (Supplementary Figure S12a). Remarkably, ZNF506 was found to be most significantly overexpressed in AML, although no significant mutation tendency was observed (Supplementary Figure S12a, b). Interestingly, upon evaluating the expression profiles of all 348 human KZFPs in AML patients, we detected a significant increase (fold change > 1, *P*-value < 0.05) in the expression of 95 KZFPs, ranking third among all cancer types (Supplementary Figure S12c). Notably, the fold change of ZNF506 expression ranks second among these KZFPs (Figure 6A).

Given the crucial role of KZFPs in repressing the transcriptional activities of repeat elements, it is possible that the overexpression of multiple KZFPs in AML could substantially affect the expression of repeat elements. To investigate this hypothesis, we re-analyzed RNA-seq data from previous studies of AML-leukemic blast cells and normal CD34+ cells (54,55). Our analysis revealed that ZNF506-targeted ERVPs were significantly down-regulated in AML-leukemic blast cells, consistent with the overall trend of repeat element expression in these cells (Figure 6B; Supplementary Figure S12d) (54). It is noteworthy that another plausible explanation for the above observations is that a particular cell type or state within AML cancer may overwhelm the sample, leading to the detection of transcriptomic patterns reflective of that cell type rather than an up-regulation of KZFPs or down-regulation of ERVs.

In AML NB4 cells, we observed enrichment of H3K9me3 signals in ERVP subfamilies targeted by ZNF506 (Supplementary Figure S12e). Additionally, we observed that the SETDB1 KO in THP-1 AML cells could partially rescue the phenotype, leading to an increase in the expression of ERVPs repressed by ZNF506 binding (Figure 6B; Supplementary Figure S12e) (55). Furthermore, *ZNF506* KO in NB4 cells also resulted in elevated expression of the targeted ERVPs (Figure 6C). To validate this finding, we infected *ZNF506* WT and KO NB4 cells with pMX-GFP pseudoviruses, and analyzed the cells via FACS. Our results revealed that *ZNF506* KO NB4 cells were more susceptible to viral infection compared with WT NB4 cells (Figure 6D; Supplementary Figure S12f).

An elevated expression of SETDB1 in numerous types of cancers, leading to increased repression of repeat elements has previously been reported (55). Clinical evidence from AML patients has demonstrated that higher repeat element expression is associated with improved survival rates (54). Using gene expression and survival data from AML patients available in GEPIA, we classified patients into two groups based on their KZFP expression levels. Our analysis revealed that patients with lower KZFP expression had better disease-free survival compared with those with higher expression (Figure 6E). These findings highlight the crucial role of regulating repeat element expression for cell regulation and immunity. In AML cells, high KZFP expression, such as ZNF506, may disrupt repeat-mediated immune processes, thereby contributing to disease development (54,55) (Figure 6F). Thus, ZNF506 may serve as a biomarker for AML prognosis.

## DISCUSSION

In this study, we identified the PBS-Pro-binding protein ZNF506 in humans. Similar to mouse ZFP809, human ZNF506 specifically targeted the PBS-Pro sites in the genome and subsequently recruited heterochromatic modifications on ERVP subfamilies for transcriptional repression.

The ZNF506 gene is unique to primates and is located within a rapidly evolving KZFP gene cluster on chromosome 19 with high protein similarities (Supplementary Figure S9c). Thus, it is likely that ZNF506 evolved from the older gene nearby, ZNF90, to protect against threats from ERVs harbored in the genome or invasion by exogenous retroviruses by targeting PBS-Pro sequences. Our analysis also revealed the presence of ERV subfamilies with PBS-Pro elements in several species, including *O. aries*, *A. melanoleuca*, *B*.*musculus*, *M*.*lucifugus* and *P. leogenomes* (Supplementary Figure S10). However, we did not find a homologous protein to ZNF506 or ZFP809 in these species (Supplementary Figure S11). We speculated that the PBS-Pro-utilizing exogenous retroviruses invaded different ancestors of these species after their divergence, including mice and humans, thus leading to the independent evolution of completely different KZFPs for host defense mechanisms.

PBS is a relatively conserved element that plays a pivotal role in the replication of exogenous retroviruses. Studies have identified several KZFPs with the capacity to repress retroviruses containing PBS sequences. These include ZNF417/ZNF587 that target PBS-Lys utilized by HIV-1 (31), and ZNF506, which targets PBS-Pro utilized by HTLV. However, the discovery of restriction factors for other retroviruses, such as Rous sarcoma virus (RSV) that utilizes PBS-Trp (56,57), remains elusive. Moreover, the human genome contains a substantial number of ERVs containing PBS-Trp (24), implying the potential existence of specific KZFPs that may target these ERVs. Nevertherless, no KZFPs demonstrating similar functions have been discovered in the high-throughput KZFP ChIP-seq data (16). Thus, the exploration of such KZFPs constitutes a vital focus for subsequent research in this field.

Previous studies suggest that the insertion of transcriptional transposons near critical genes can lead to the development of certain cancers, either by inducing mutations or by altering gene expression (58,59). Our study provides new insights in this regard. We have identified a significant correlation between KZFPs and leukemia. In AML patients, we observed a significant up-regulation of ZNF506 and other KZFPs, which led to the repression of targeted repeat elements (Figure 6A, B). The increase in KZFP expression may affect the immune sensitivity of cells, allowing cancer cells to escape immune detection (Figures 6F). Additionally, accumulating evidence indicates that SETDB1 inhibitors could be potentially utilized in the treatment of AML disease (55), consistent with the observed high activity of KZFPs in AML cells (Figure 6B). Therefore, elucidating the functions of other overexpressed KZFPs can not only deepen the understanding of the molecular mechanisms underlying diseases such as AML, but can also provide potential targets for subsequent treatments for these diseases. Importantly, our findings indicate that the expression of KZFPs and repeat elements is associated with the disease-free survival rate of AML patients (Figure 6E), suggesting its potential as a biomarker for assessing the effectiveness of AML therapy.

Previous studies have provided in-depth insights into the function of ZFP809 in repressing MLV by specifically targeting PBS-Pro sites (10). For humans, infection by the PBS-Pro-containing leukemia-inducing virus HTLV-1 carries a certain risk of causing adult T-cell leukemia/lymphoma (ATLL) (60–62). However, different from AML that stems from bone marrow stem cells and predominantly affects myeloid cells such as granulocytes, red blood cells and platelet precursors in the hematopoietic system, ATLL is a T-cell leukemia/lymphoma with distinct pathogenic mechanisms (60,63). Interestingly, previous studies on T-cell leukemia have revealed that ZNF506 expression is significantly suppressed in ATLL cases caused by HTLV-1 infection, compared with T-cell leukemia arising from non-HTLV infection (64). This observation may reflect a potential role for ZNF506 in the pathogenesis of ATLL.

Taken together, our study identified ZNF506 as the human PBS-Pro-binding protein that recruits heterochromatic modifications to silence the transcription of ERVP subfamilies and PBS-Pro-utilizing exogenous retroviruses. The evolutionary traits of ZNF506 and its relationship with ERVP subfamilies suggest a constant struggle between KZFPs and ERVs. Furthermore, the interplay between repeat elements and KZFPs may have a significant impact on the pathogenesis and progression of diseases.

## Supplementary Material

gkad731_Supplemental_files

## Data Availability

All of the data were analyzed with standard programs and packages as detailed. The sequencing data generated in this study can be found at GEO: GSE209826 (https://www.ncbi.nlm.nih.gov/geo/query/acc.cgi?acc = GSE209826). The following public ChIP-seq datasets were used: ZNF506 [Sequence Read Archive (SRA) accession: SRR5197160], ZFP809 (SRA accession: SRR1188176), ZNF253 (SRA accession: SRR14103920), ZNF682 (SRA accession: SRR5197216), ZNF93 (SRA accession: SRR5197268), ZNF90 (SRR5197267), ZNF486 (SRR5197156), ZNF737 (SRR5197228), ZNF626 (SRR5197202) and ZNF765 (SRA accession: SRR5197234), human ESC KAP1 (SRA accession: SRR3178875), human ESC H3K9me3 (SRA accession: SRR8983692), human ESC H3K27ac (SRA accession: SRR8983687), human ESC ATAC-seq (SRA accession: SRR7942724), human ESC DNMT3A (SRA accession: SRR14005187), human ESC DNMT3B (SRA accession: SRR14005189), human ESC RNA-seq (SRA accession: SRR5143952), SETDB1 WT/knockdown H3K9me3 (SRA accession: SRR6653350/SRR6653351), SETDB1 WT/knockdown H3K4me1 (SRA accession: SRR6653354/SRR6653355), NB4 H3K9me3 (SRA accession: SRR032061), repeat expression in AML (GEO database accession: GSE175701), SetDB1 WT/knockdown in AML (GEO database accession: GSE103409).
