# Supplementary material for: Unraveling the role of ZNF506 as a human PBS-pro-targeting protein for ERVP repression
Source: Nucleic Acids Res. 2023 Sep 11;51(19):10309–25. doi: 10.1093/nar/gkad731 (PMC10602909; doi:10.1093/nar/gkad731)
Supplement: gkad731_Supplemental_files [file gkad731_supplemental_files.zip › Supplementry Figures.pdf]

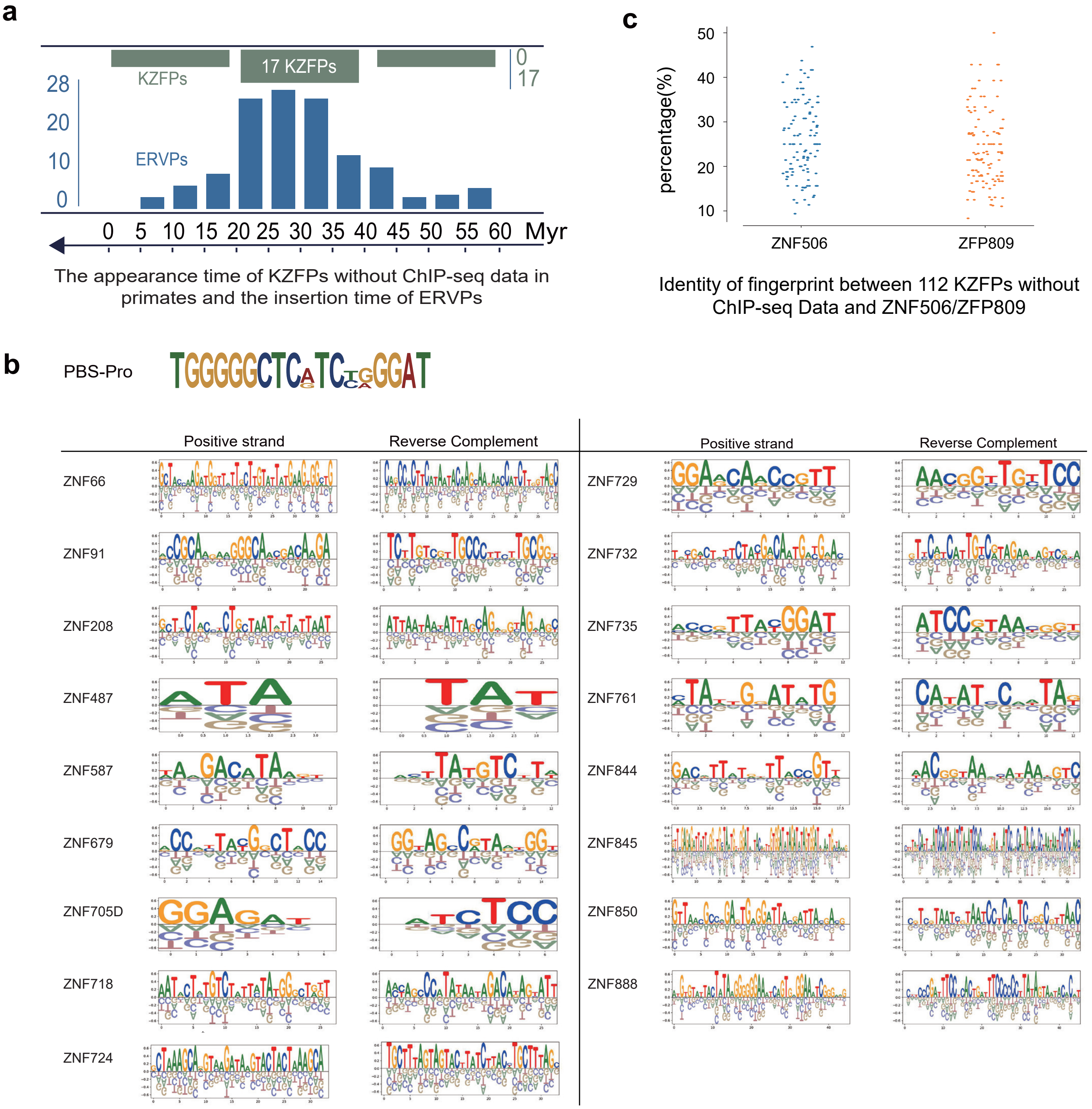

Figure S1.Screening KZFPs Targeting PBS-Pro in Humans.

a, Analysis of the insertion timing of ERVPs into the human genome and the emergence of the 112 KZFPs without ChIP-seq data in primates. The most recent common ancestor (MRCA) method was employed to determine the age of KZFPs, pinpointing those that coincided with ERVPs. 17 KZFPs lacking ChIP-seq data were identified to display evolutionary association with ERVPs. **b,** Predicted DNA-binding motifs based on the Zinc-finger code of the 17 KZFPs. **c,** Correlation between the fingerprint Identities of 112 KZFPs lacking ChIP-seq Data and ZNF506/ZFP809.

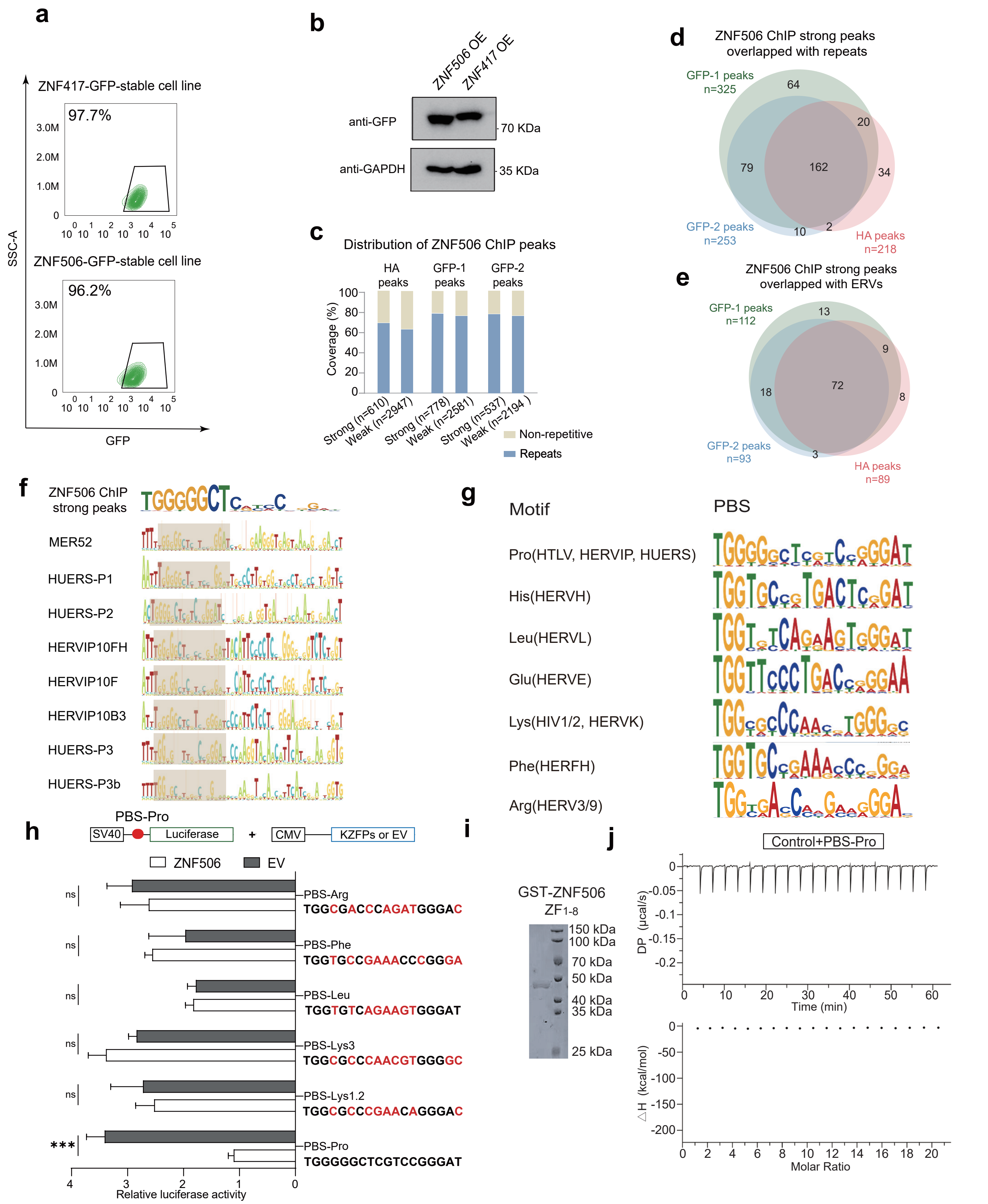

Figure S2. ZNF506 specifically binds to the PBS-Pro sequences.
a–b, FACS analysis (a) and western blot (b) showing 293T cells stably overexpressing GFP-tagged ZNF506 or ZNF417. OE, overexpression. **c,** Histogram showing the three groups of ZNF506 ChIP-seq peaks overlappping in the genome. **d,** Venn diagram showing the overlap between three groups of strong ZNF506 ChIP-seq peaks at repeat regions. **e,** Venn diagram showing the overlap between three groups of strong ZNF506 ChIP-seq peaks at ERV regions. **f,** Comparision of the DNA binding motif of ZNF506 with the first 50 nt of ERVP family. **g,** Comparison of PBS sequences from major human ERVs. **h.** Relative luciferase activity of 293T cells overexpressing ZNF506 or an empty vector (EV) and SV40 promoter-driven luciferase plasmid containing different PBS sequences (right). The nucleotides different from the PBS-Pro sequences are marked in red. t test: error bars indicate standard deviation. **i,** GST fusion ZNF506-ZF1-8 resolved on a 10% SDS-PAGE gel and visualized by Coomassie blue stain. **j,** Isothermal titration calorimetry (ITC) binding assay of PBS-Pro double-stranded oligonucleotides titrated with blank protein.

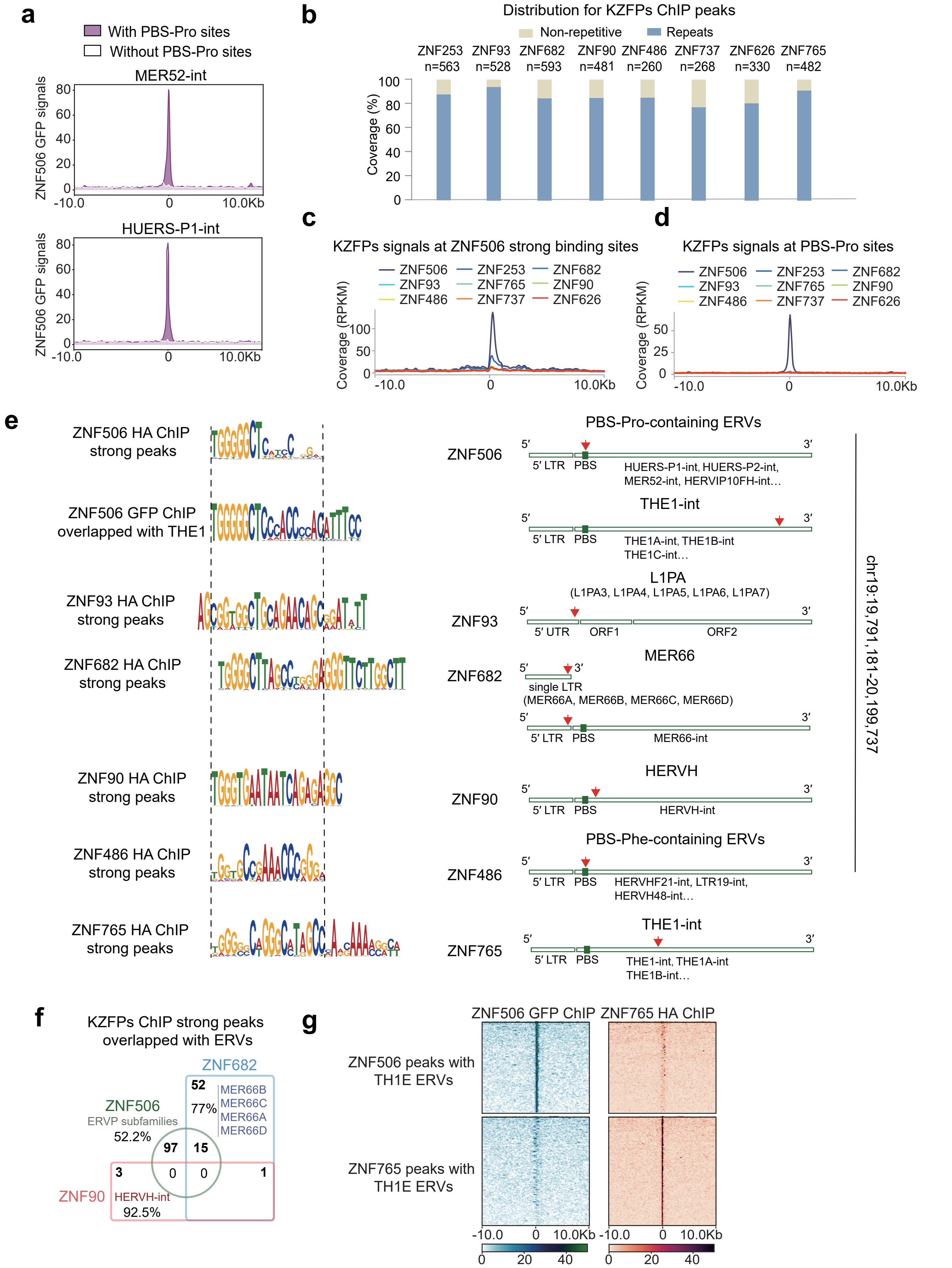

Figure S3. Functional diversity of KZFPs located close to the ZNF506 gene locus.
a, Profile showing ZNF506-GFP signals at ERVP subfamilies with or without PBS-Pro sites. **b,** Histogram showing different KZFP high confidence ChIP-seq peaks overlapped in the genome. **c,** Profiles showing different KZFP signals at strong binding sites of ZNF506. **d,** Profiles showing different KZFP signals at PBS-Pro sites. **e,** ChIP-seq motifs of different KZFPs and ZNF506 at THE1 subfamilies (left), and genome-wide KZFPs binding subfamilies (right). Red arrows indicate specific locations of subfamilies bound by KZFPs. The ChIP-seq data for HA-tagged ZNF93, HA-tagged ZNF682, HA-tagged ZNF90, HA-tagged ZNF486 and HA-tagged ZNF765 were from Imbeault et al. **f,** Venn diagram showing the overlap between ZNF506, ZNF90, and ZNF682 ChIP-seq peaks at ERV regions. The percentages represent the proportion of the marked subfamilies' peak numbers relative to the total number of strong peaks. **g,** Heatmap showing ZNF506 and ZNF765 ChIP-seq data at THE1 subfamilies, which are bound by these two KZFPs.

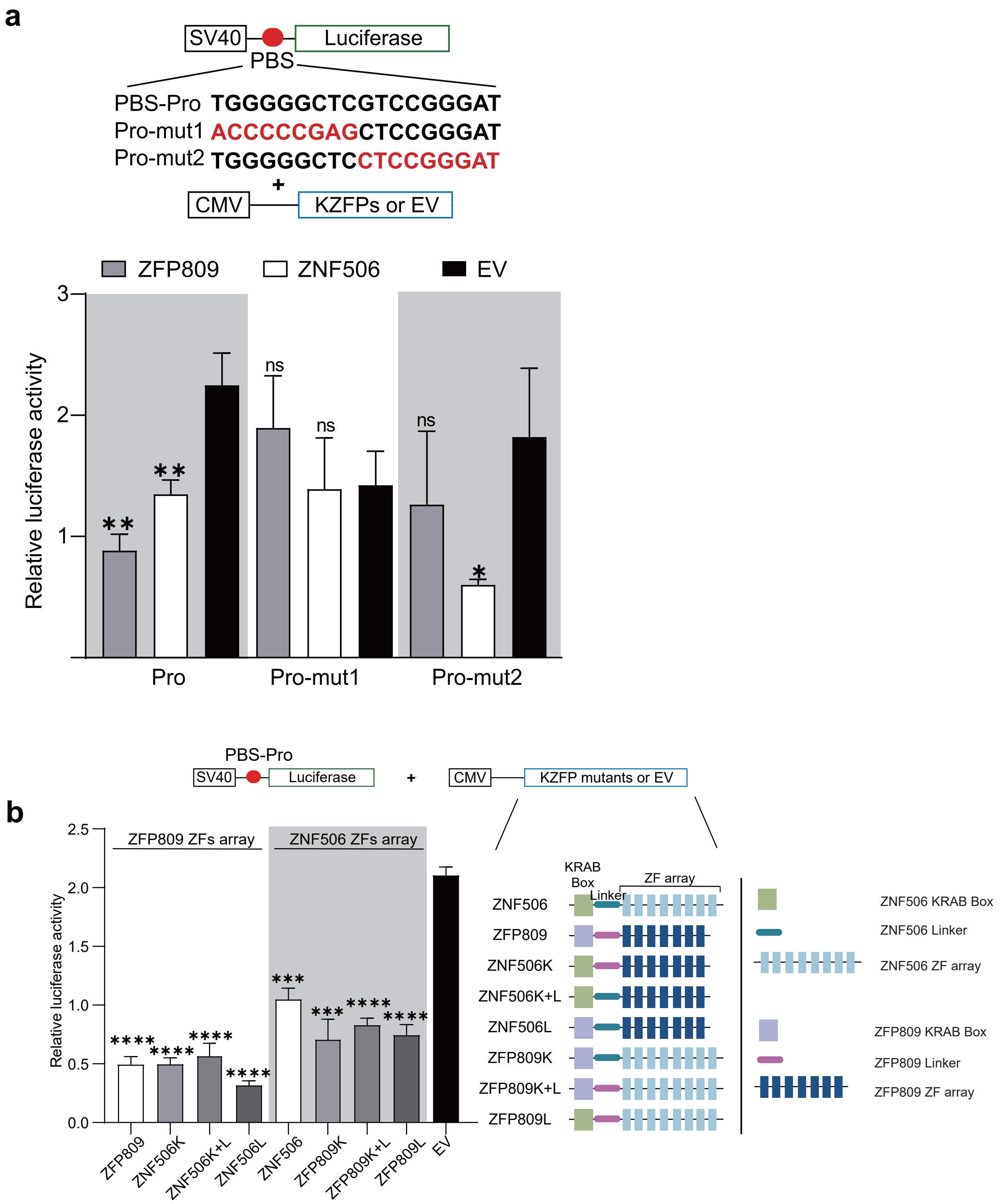

Figure S4. Differences in ZF arrays between ZNF506 and ZFP809.
a, Relative luciferase activity of 293T cells overexpressing ZNF506, ZFP809, or an empty vector (EV), and an SV40 promoter-driven reporter containing PBS-Pro sequence or mutants. t test: error bars indicate standard deviation. *$p < 0.05$, **$p < 0.01$, ns $p > 0.05$; n = 3. **b,** Relative luciferase activity of 293T cells overexpressing ZNF506, ZFP809, or KZFP mutant plasmids (right) by exchanging two KZFP domains and an SV40 promoter-driven reporter containing a PBS-Pro sequence. t test: error bars indicate standard deviation. ***$p < 0.001$, ****$p < 0.0001$, ns $p > 0.05$; n = 3.The combination of KRAB box, Linker and ZF array is explained on the right.

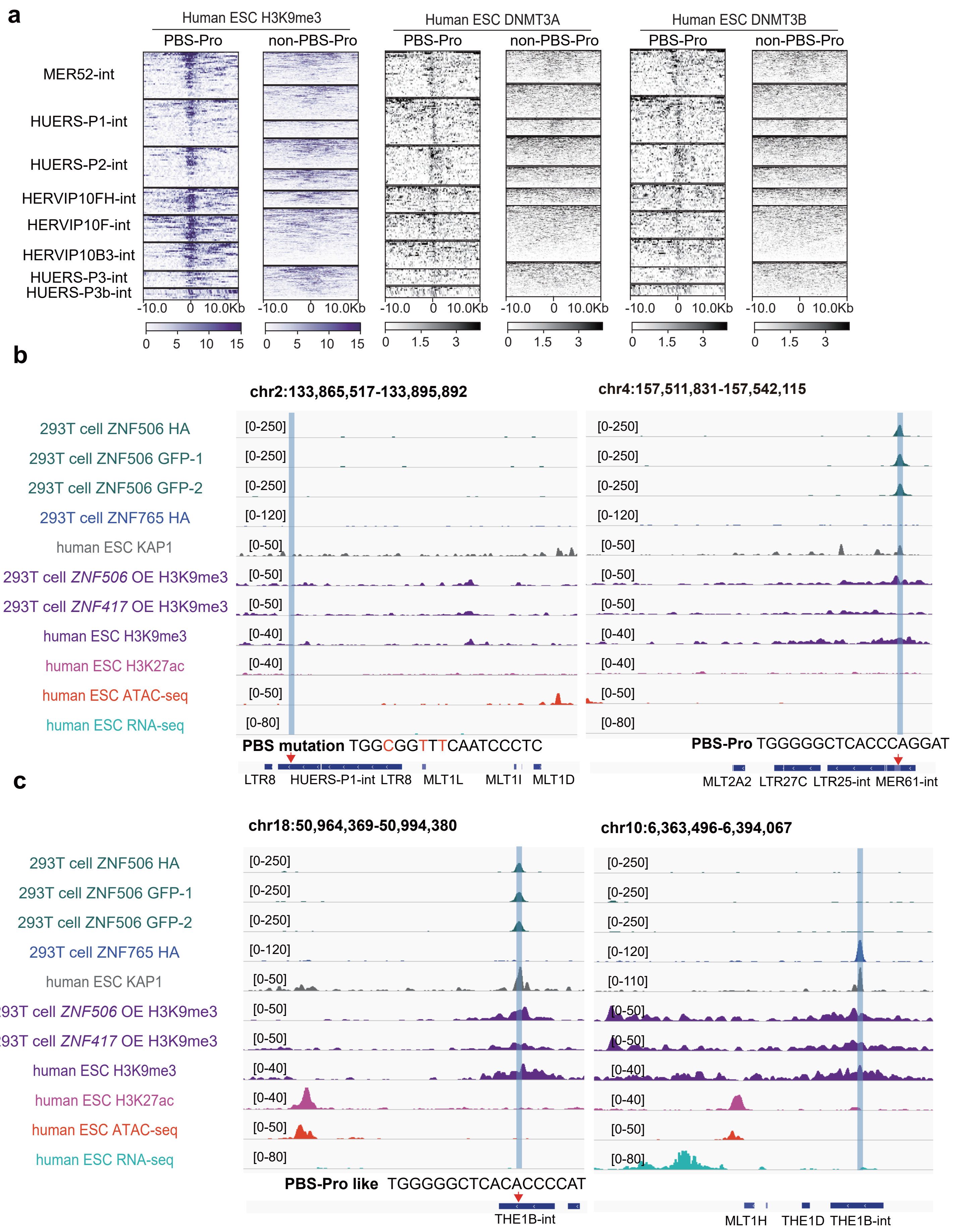

Figure S5. ZNF506 binds to ERV subfamilies with Pro-PBS-like sequences.
a, Heatmap showing ChIP-seq data for hESC H3K9me3, DNMT3A, and DNMT3B at ERVP subfamilies with or without PBS-Pro sites. **b–c,** ChIP-seq signals for ZNF506, ZNF765, KAP1, H3K9me3, H3K27ac, as well as ATAC-seq and RNA-seq signals at ERV subfamilies. The nucleotides different from the first 9 nt of the PBS-Pro sequences are highlighted in red.

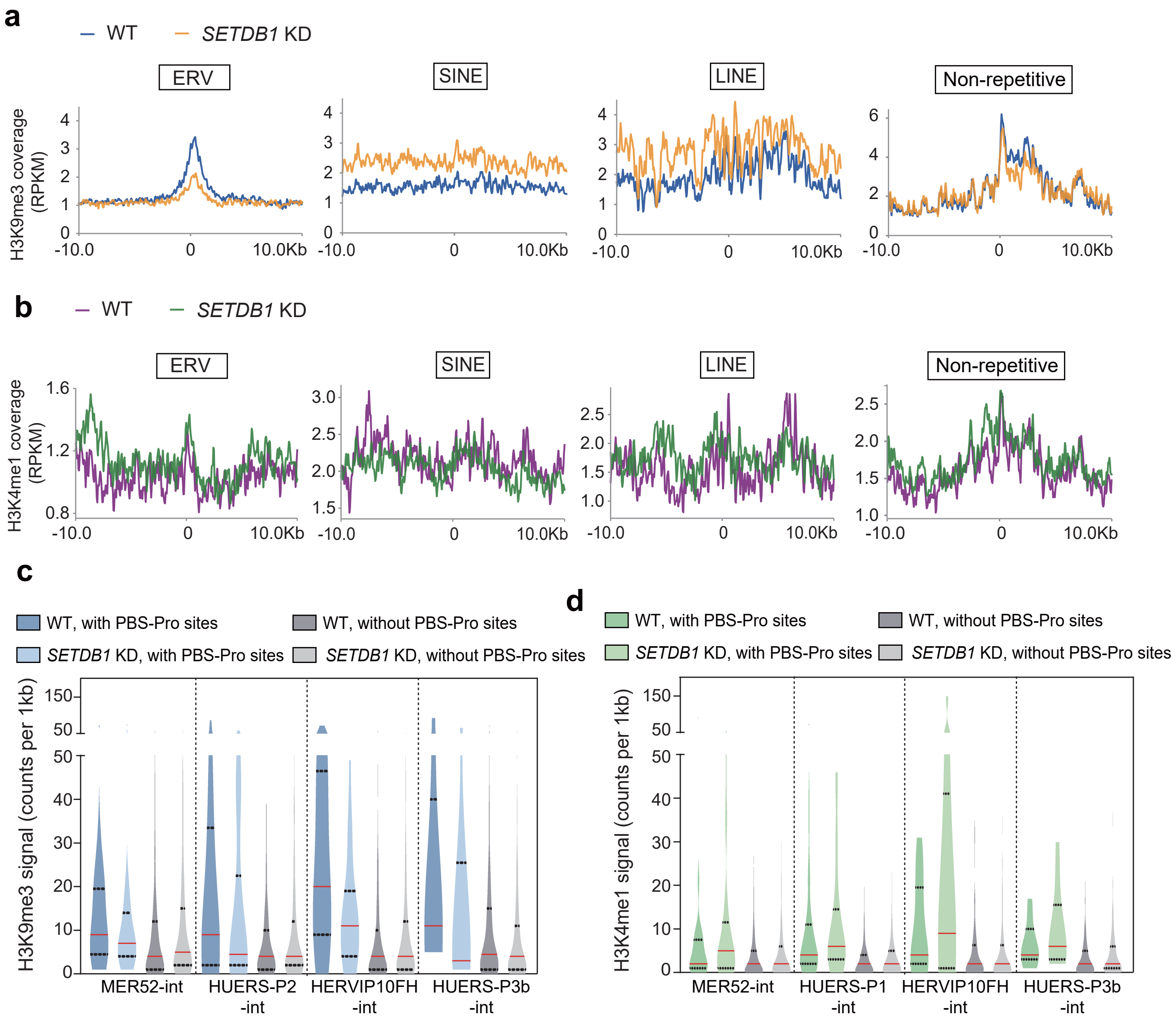

Figure S6. Changes in histone modifications at ERVP subfamilies with PBS-Pro sites following SETDB1 knockdown.

a–b, Profiles showing H3K9me3 (a) and H3K4me1 signals (b) at ZNF506 binding regions before and after SETDB1 knockdown (KD) in melanoma cell line. **c–d,** Violet plots showing H3K9me3 (c) and H3K4me1 signals (d) at ERVP subfamilies with or without PBS-Pro sites before and after SETDB1 KD in the melanoma cell line.

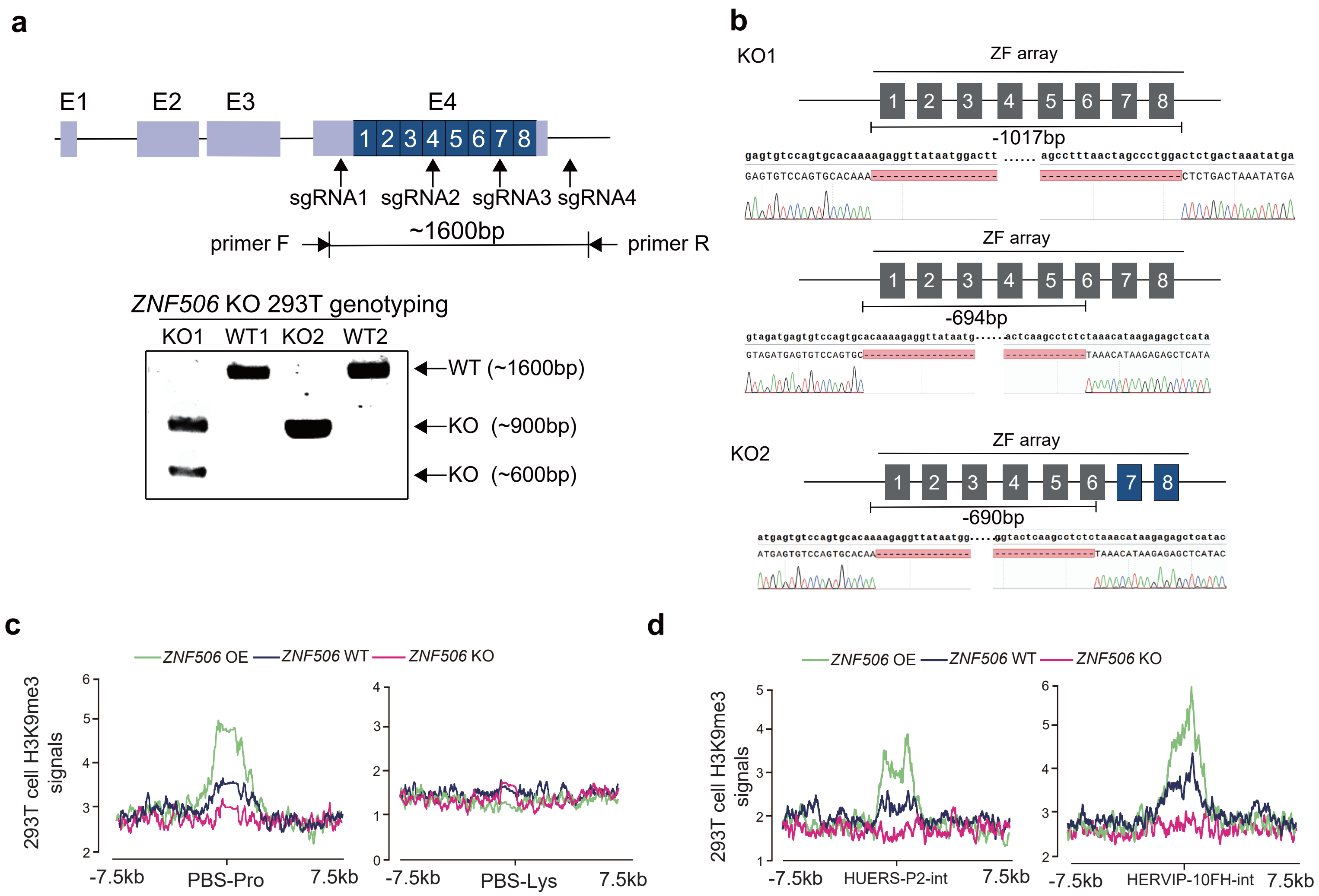

Figure S7. Reduction of H3K9me3 modifications in ERVP subfamilies following ZNF506 knockout (KO). **a–b,** Genotyping PCR analysis (a) and subsequent sequencing results (b) to confirm deletion of ZNF506 alleles in 293T cells. **c,** Profiles showing H3K9me3 signals at PBS-Pro sites and PBS-Lys in *ZNF506* OE ,WT and KO 293T cells. **d,** Profiles showing H3K9me3 signals at ERVP subfamilies with PBS-Pro sites in *ZNF506* OE , WT and KO 293T cells.

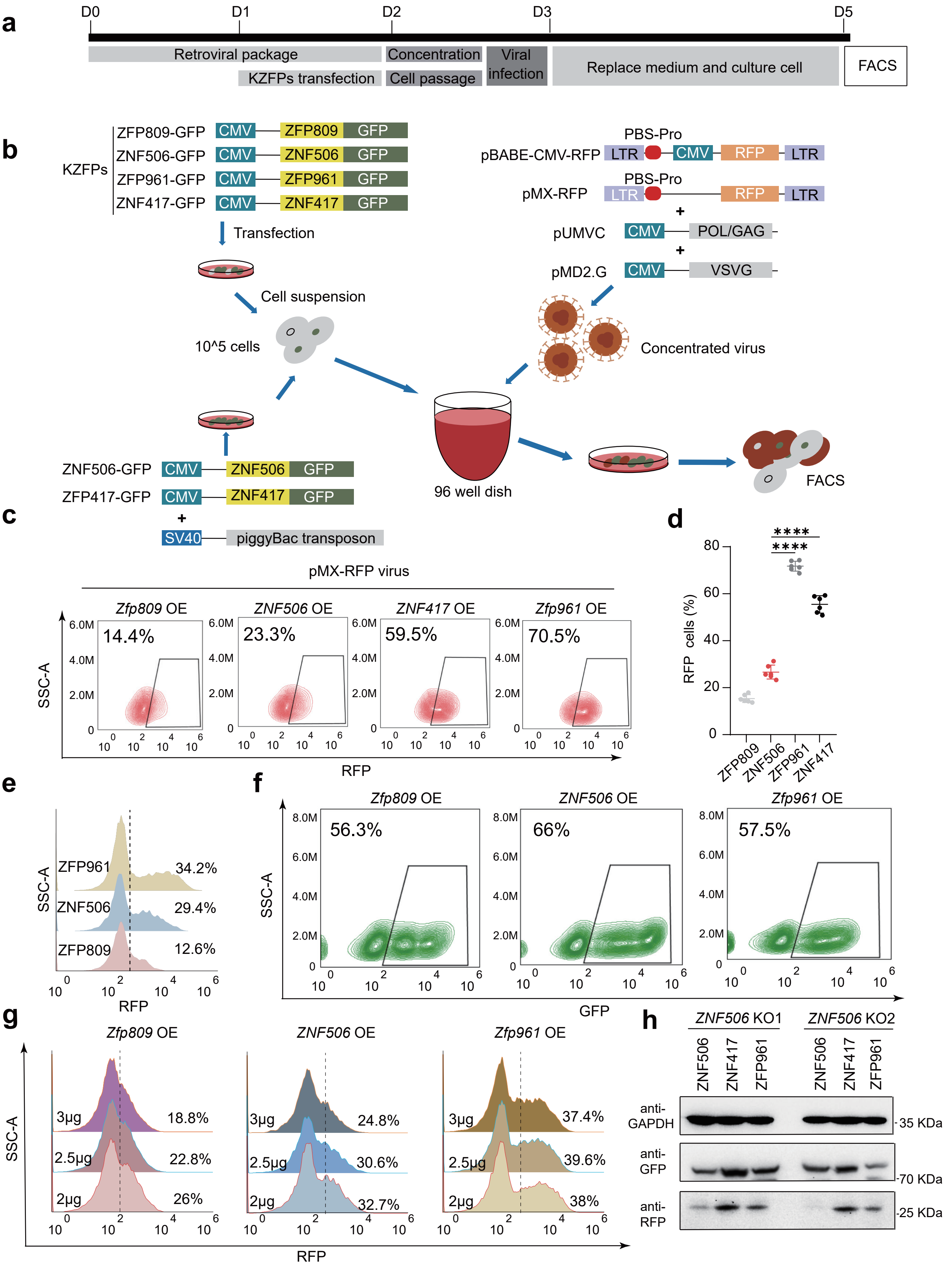

Figure S8. ZNF506 restricted the transcription of PBS-Pro-utilizing pseudoviruses.
a, Graphical timeline of the virus infection experimental procedure. **b,** Schematic showing an overview of the PBS-Pro-utilizing pseudoviral infection repression assay. The pseudoviral plasmid pMX-RFP or pBABE-RFP, together with their respective packaging plasmids, was transfected into 293T cells to generate viral particles, which were then infected in 293T cells overexpressing KZFPs. Cells were harvested and subjected to FACS analysis 48 hours after infection. **c–d,** FACS analysis (c) and statistical plot (d) showing the pMX-RFP viral infection rate in 293T cells overexpressing GFP-tagged ZNF809, ZNF506, ZNF417, or ZNF961. t test: error bars indicate SD; ****$p < 0.0001$, n = 6. **e–f,** FACS analysis showing RFP expression in 293T cells co-transfected with the pMX-RFP vector and ZFP809, ZNF506, or ZFP961 as control vectors (e) and the expression of GFP-fused KZFPs (f). **g,** FACS analysis showing RFP expression in 293T cells co-transfected with the pMX-RFP vector and different amounts of KZFP plasmids. **h,** Western blot showing RFP expression in ZNF506 KO 293T cells co-transfected with the pMX-RFP vector and ZNF506, ZNF417, or ZFP961 plasmids.

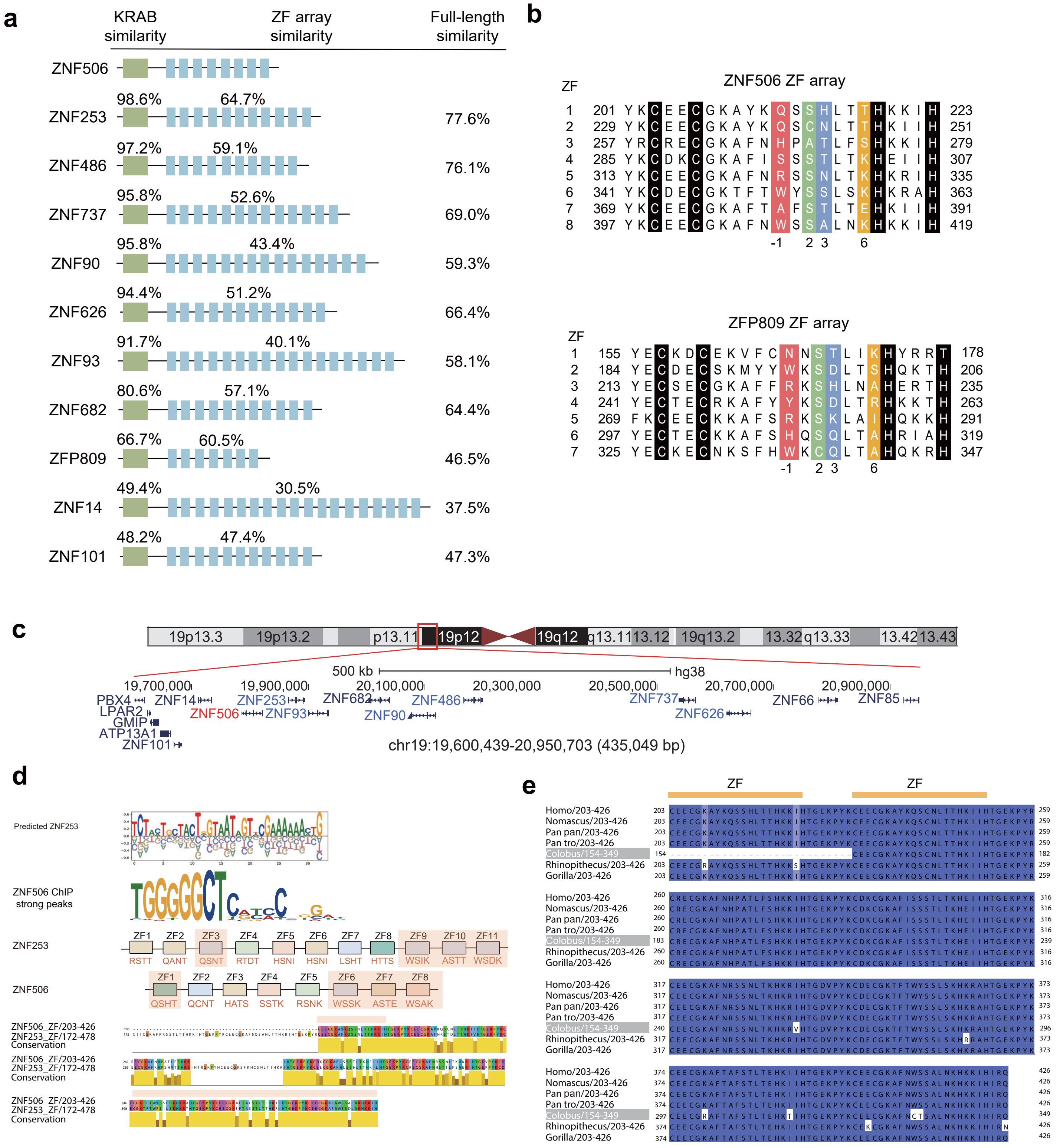

Figure S9. ZNF506 is conserved during primate evolution.
a, Graphical representation of protein pairwise sequence alignments between ZNF506 and mouse ZFP809, as well as KZFPs located close to the ZNF506 gene locus, including KRAB domains, ZF arrays, and the full-length proteins. **b,** Schematic depiction of ZNF506 and ZFP809. The four Zn-coordinating residues of each finger are highlighted with white letters against black, and the four key amino acids of their ZF array (−1, 2, 3, 6) are in colors. **c,** Annotated gene transcripts in the KZFP gene cluster containing ZNF506 (red) on chromosome 19 (chr19), hg38. Data are from the “UCSC Genes” track in the UCSC genome browser. **d,** Comparison of DNA binding motifs, key ZF amino acids and tandem ZF domains of ZNF506 and ZNF253. **e,** Alignment of ZF regions among ZNF506 orthologs.

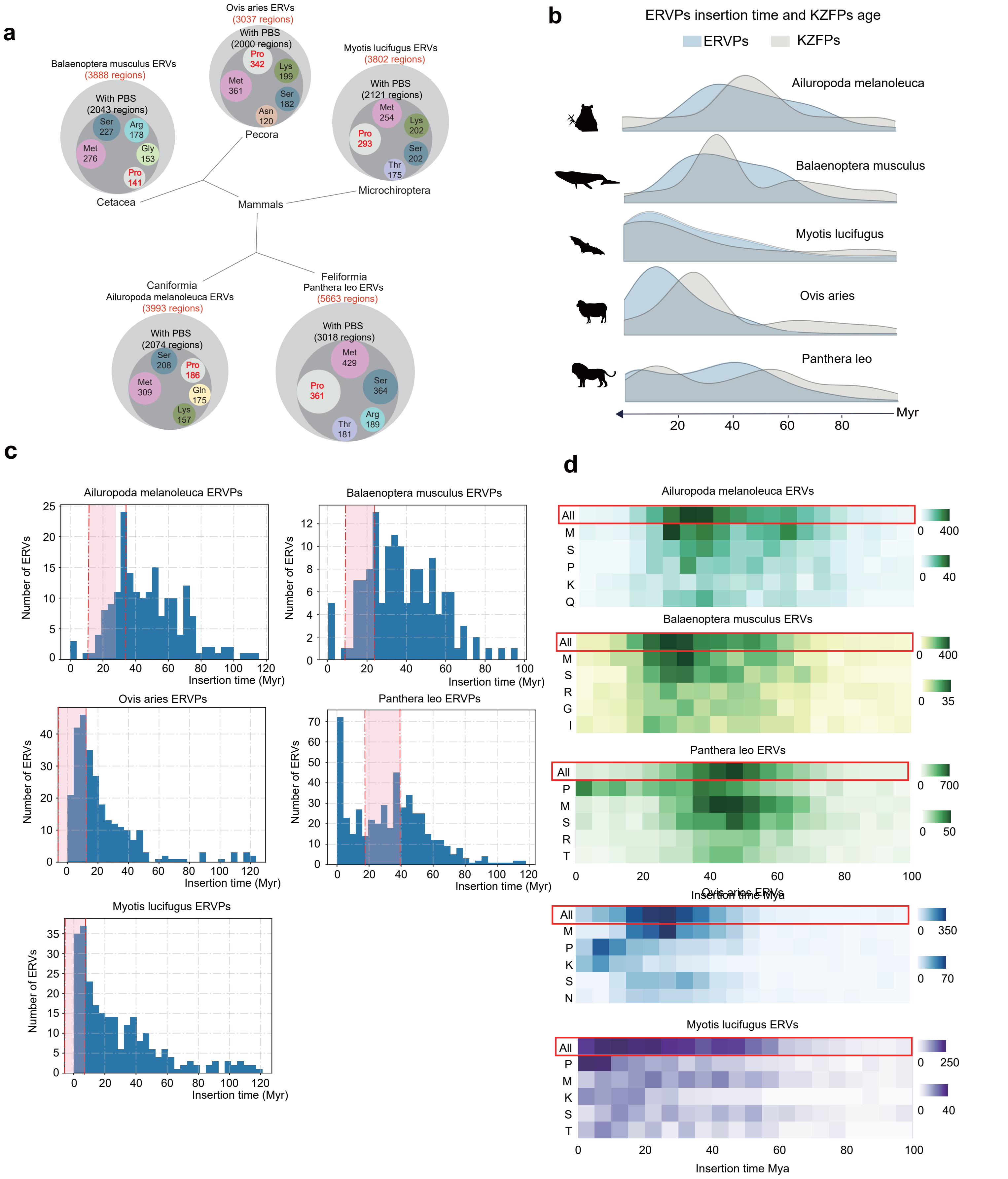

Figure S10. Insertion time distribution of ERVPs subfamilies in the five species.

a, Classification of ERVs by the type of PBS sequences in Ovis aries, Ailuropoda melanoleuca, Balaenoptera musculus, Myotis lucifugus, and Panthera leo according to the calculation results of RetroTrector. **b,** Correspondence between the emergence time of KZFPs and the insertion time of ERVPs subfamily in these five species. Myr, million years.**c,** Insertion time distribution of ERVPs subfamilies in Ovis aries, Ailuropoda melanoleuca, Balaenoptera musculus, Myotis lucifugus, and Panthera leo. **d,** Distribution of the insertion time of each ERVs subfamily in the five species using PBS as the classification standard. The darker a certain color block is, the more ERVs are inserted in the time period. For the sake of display, the color bar of the overall ERVs insertion time and the insertion time of each PBS subfamily is different.

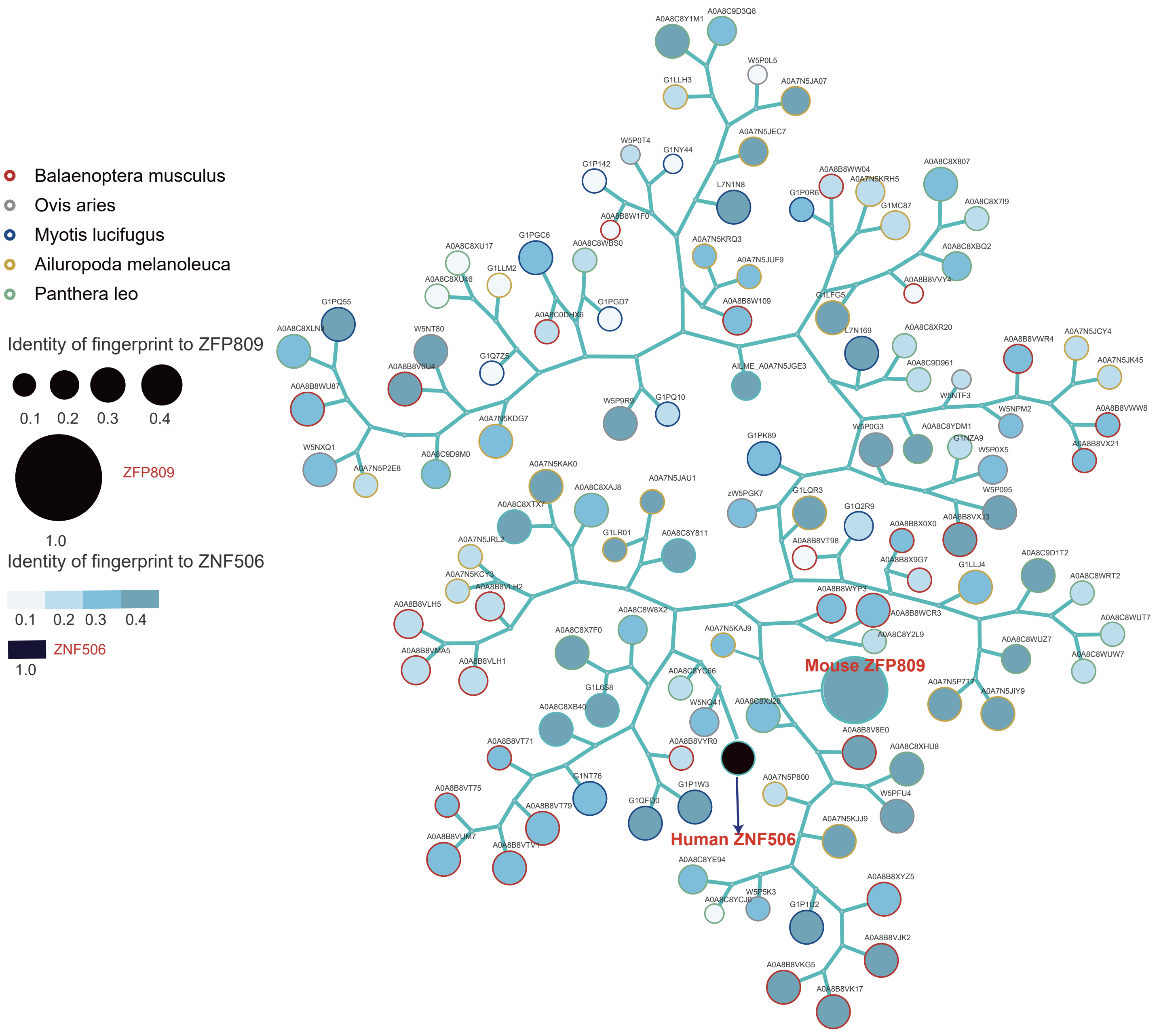

Figure S11. ZNF506 and ZPF809 have no homologous proteins in other species.
Comparison of human ZNF506, mouse ZFP809 and KZFPs that may have PBS-Pro binding capacities in these five species. The evolutionary tree represents the evolutionary distance between these KZFPs, the color depth represents the similarity of fingerprint with ZNF506, and the size of the circle represents their similarity of fingerprint with ZFP809.

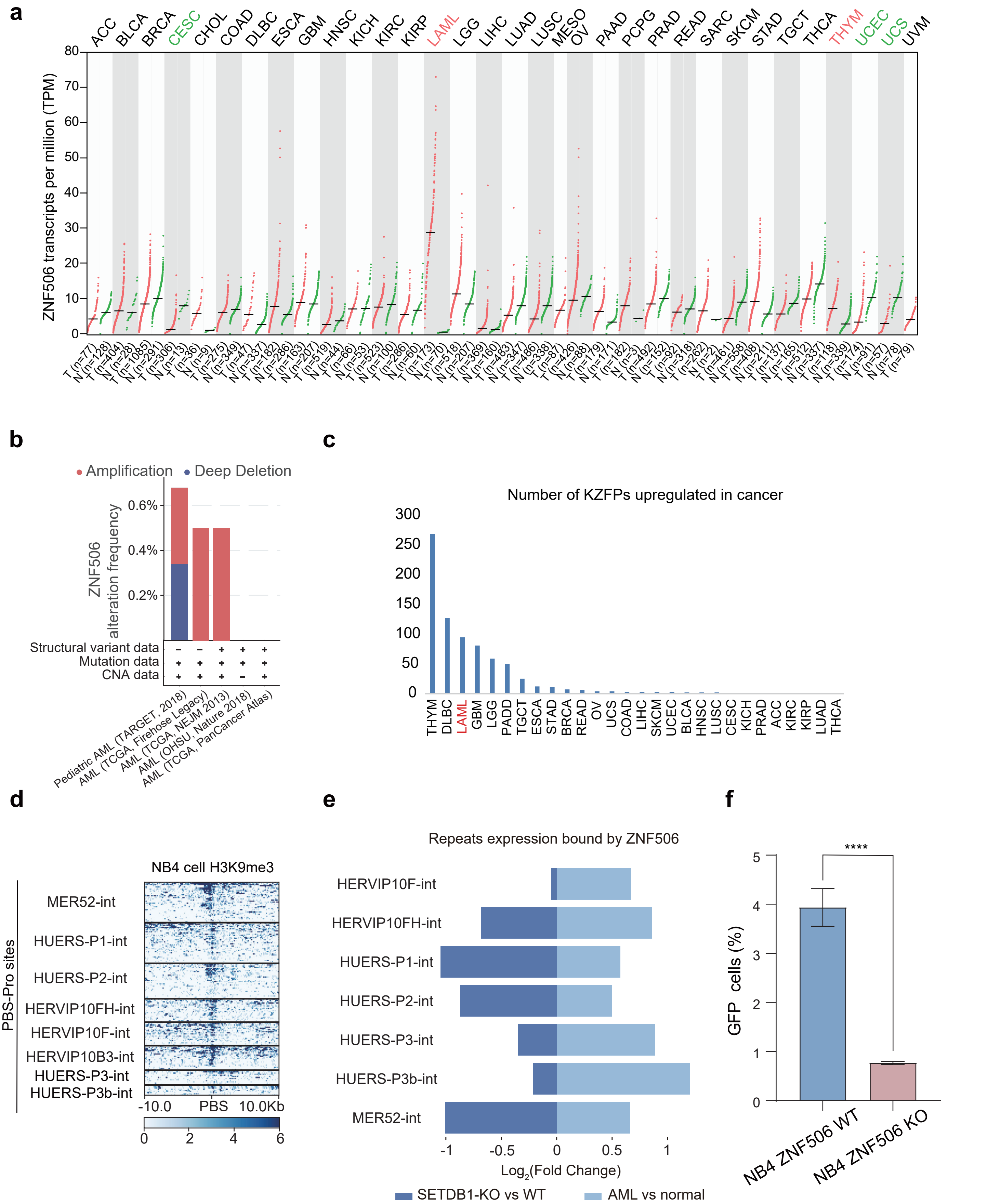

Figure S12. Association of ZNF506 with a range of cancers.

a, Profile showing ZNF506 expression in tumor samples of various cancers compared with normal samples. Abbreviations refer to The Cancer Genome Atlas (TCGA) database. **b,** Histogram showing the frequency of ZNF506 gene alterations in AML patients. **c,** Number of KZFPs with increased expression across diverse cancers. **d,** Heatmap showing the presence of H3K9me3 signals at ERV-associated PBS-Pro sites in NB4 cells. The 20 kb regions are displayed with the PBS-Pro sequence at the center of the peak regions. **e,** Changes of ERVP expression in AML cells and SETDB1 KO AML cells. **f,** pMX-IRES viral infection rate in NB4 cells before and after ZNF506 KO. t test: error bars indicate standard deviation. ****$p < 0.0001$, $n = 6$.
